# AI-Based System for Analysis of Electron Microscope Images in Glomerular Disease

**DOI:** 10.1001/jamanetworkopen.2025.34985

**Published:** 2025-10-07

**Authors:** Pengcheng Ma, Jinbang Li, Zhengyu Zhang, Weihao Qiu, Danyi Li, Jing Wang, Bingbing Li, Shujing Guo, Jin Zhang, Zhijian Cen, Jian Geng, Xiangsheng Huang, Xiaolei Xue, Aihetaimu Aimaier, Huanjiao Liu, Minyi Liang, Hao Chen, Qifeng Jiang, Xiaoyan Su, Tianjun Guan, Yu Tong, Weiyuan Lin, Li Liu, Jun Xu, Jie Lin, Yaping Ye, Li Liang

**Affiliations:** 1 Big Data Center, Nanfang Hospital, Southern Medical University, Guangzhou, China; 2 Department of Pathology, Nanfang Hospital, School of Basic Medical Sciences, Southern Medical University, Guangzhou, China; 3 Guangdong Province Key Laboratory of Molecular Tumor Pathology, Guangzhou, China; 4 Jinfeng Laboratory, Chongqing, China; 5 Department of Pathology, Ganzhou Hospital, Guangdong Provincial People’s Hospital, Ganzhou, China; 6 Suzhou Hospital, Affiliated Hospital of Medical School, Nanjing University, Suzhou, China; 7 Cognitive Intelligence Lab, Xiongan Institute of Innovation, Chinese Academy of Sciences, Xiongan, China; 8 Department of Computer Science and Engineering, The Hong Kong University of Science and Technology, Hong Kong, China; 9 Department of Chemical and Biological Engineering, The Hong Kong University of Science and Technology, Hong Kong, China; 10 Department of Renal Pathology, Guangzhou Huayin Medical Laboratory Center, Guangzhou, China; 11 Department of Nephrology, Dongguan Tungwah hospital, Dongguan, China; 12 Department of Nephrology Zhongshan Hospital Affiliated to Xiamen University, Xiamen, China; 13 Department of Nephrology, The People’s Hospital of Gaozhou, Maoming, China; 14 Department of Nephrology, Fujian Medical University 2nd Affiliated Hospital, Fuzhou, PR China; 15 School of Public Health, Southern Medical University, Guangzhou, China

## Abstract

**Importance:**

Kidney biopsy pathology via transmission electron microscopy (TEM) is essential for diagnosing glomerular diseases, offering critical information on glomerular basement membrane (GBM) thickness, foot process (FP) number, and electron-dense deposits (EDDs). These tasks are laborious and time-consuming.

**Objective:**

To develop and validate an artificial intelligence (AI) diagnostic system, TEM image–based AI-assisted device (TEM-AID), that accurately segments and measures glomerular ultrastructures (including the GBM, FPs, and EDDs) and determines glomerular disease subtypes using TEM images.

**Design, Setting, and Participants:**

This diagnostic study used a large, multicenter cohort including 160 727 TEM images from 31 670 patients with chronic kidney disease across 6 medical centers from January 2021 to December 2023. TEM-AID was trained and validated on 26 650 patients from 1 center and tested externally on 5020 patients (5 test sets) plus a human-AI test set (454 patients representing 7 glomerular disease subtypes). Data were analyzed from January to December 2024.

**Exposures:**

TEM-AID integrates 4 modules. Segmentation combined YOLO-v8 detection, segment anything model, and human-in-the-loop refinement to segment GBMs, podocyte FPs, and EDDs. Measurement quantified GBM thickness, FP fusion degree, and EDD deposition sites. Classification used least absolute shrinkage and selection operator–selected deep learning and statistical features with a stacking classifier to diagnose 7 glomerular disease subtypes: immunoglobin A nephropathy, membranous nephropathy, lupus nephritis, diabetic nephropathy, minimal change disease, mesangial proliferative glomerulonephritis, and thin basement membrane nephropathy.

**Main Outcomes and Measures:**

Outcomes of interest were segmentation performance (mean intersection-over-union [IOU], Dice coefficient), subtype classification accuracy, area under the receiver operating characteristic curve (AUC), and human-AI diagnostic concordance.

**Results:**

A total of 31 670 patients (mean [SD] age, 43.2 [16.5] years; 17 372 [54.9%] male) contributed 160 727 TEM images for analysis. Segmentation achieved a mean (SD) IOU of 0.835 (0.062) and Dice of 0.874 (0.023). Subtype classification accuracy was 0.911 (95% CI, 0.904-0.918) in internal validation and 0.895 to 0.914 in external tests. Macro-AUC ranged from 0.972 to 0.989 across cohorts. In human-AI testing (454 patients), TEM-AID accuracy (0.886 (95% CI, 0.859-0.912]; AUC, 0.963 [95% CI, 0.937-0.989]) exceeded clinicians’ unaided performance. Clinicians’ accuracy improved by a mean (SD) of 11.7% (5.2%) when they used TEM-AID.

**Conclusions and Relevance:**

In this multicenter diagnostic study, TEM-AID precisely quantified glomerular ultrastructures and determined glomerular disease subtypes from TEM images, significantly enhancing diagnostic efficiency and accuracy. This system provides quantitative evaluation tools to support clinical pathologists in diagnostic workflows, demonstrating robust multicenter performance.

## Key Points

**Question:**

What is the performance of an artificial intelligence (AI) system for the analysis of kidney transmission electron microscopy (TEM) images for diagnosing glomerular disease?

**Findings:**

This diagnostic study including 160 727 images from 31 670 patients across 6 health centers describes the development and validation of TEM-AID, an AI-based system to diagnose glomerular disease. TEM-AID accurately segmented glomerular structures and directly predicted glomerulonephritis subtypes, achieving high internal diagnostic performance and demonstrating consistent external validation across 5 test sets; additionally, in a comparative study involving 454 patients, TEM-AID outperformed pathologists.

**Meaning:**

This diagnostic study found that TEM-AID significantly enhanced diagnostic efficiency and accuracy in kidney pathology by providing quantitative TEM analysis tools, supporting pathologists in clinical practice.

## Teaser

This diagnostic study describes the development and validation of an artificial intelligence (AI) model using transmission electron microscopy for the diagnosis of glomerular disease.

## Introduction

Glomerular disease, an immune-mediated disease spectrum causing glomerular damage, is a primary contributor to chronic kidney disease, with increasing morbidity and mortality rates.^1,2^ Renal needle biopsy, the standard diagnostic method, requires comprehensive histopathological evaluation through light and transmission electron microscopy (TEM), immunofluorescence, and clinical data. TEM is crucial for identifying ultrastructural abnormalities, like glomerular basement membrane (GBM) architecture, electron-dense deposits (EDDs), and podocyte foot process (FP) morphology.^3,4^ Its use is imperative when light microscopy and immunopathological assessments yield ambiguous results due to subtle or atypical lesions.^5^ In 44.3% of glomerulopathies, TEM provides essential diagnostic data, highlighting its importance in glomerular disease diagnosis.^6^ Previous studies have attempted to quantify EM features to improve diagnosis and prediction of outcomes in patients with primary glomerular diseases. For instance, Zee et al^7^ identified several histologic and ultrastructural descriptors that were highly predictive of clinical outcomes in patients with minimal change disease and focal segmental glomerulosclerosis. The study by Zee et al^7^ highlighted the importance of features such as global sclerosis, segmental sclerosis, interstitial fibrosis or tubular atrophy, and novel descriptors, like adhesion, interstitial foam cells, and periglomerular fibrosis. These findings underscore the potential of quantitative EM analysis in enhancing diagnostic accuracy and prognostication. However, TEM analysis is labor-intensive, time-consuming, and often lacks consistency among nephropathologists,^8^ necessitating innovative diagnostic tools to enhance accuracy.

In recent years, artificial intelligence (AI) algorithms, exemplified by Transformers, have shown great potential in renal biopsy pathology research.^9,10^ They have made outstanding contributions in glomerular subtype prediction, as well as the detection and segmentation of GBMs, FPs, and EDDs.^11,12,13,14,15,16,17^ However, there are still existing issues at present, including insufficiently refined measurement of changes in glomerular ultrastructure and inaccurate subtype diagnosis. In addition, most current studies still focus on hematoxylin-eosin–stained images, while research on TEM images is relatively scarce.^18,19,20,21^

In this study, we developed an AI diagnostic system, TEM image–based AI-assisted device (TEM-AID), designed to achieve fully automatic segmentation and measurement of GBMs, podocyte FPs, and EDDs on TEM images, as well as accurate diagnosis of 7 different GBM lesions (glomerular disease). To validate its clinical utility, we examined whether TEM-AID surpassed nephropathologist performance in diagnostic accuracy while maintaining generalizability across diverse clinical settings. We hypothesize that TEM-AID will significantly outperform manual analysis and demonstrate robust diagnostic capabilities in multicenter validation.

## Methods

This multicenter diagnostic study was approved by the Medical Ethics Committee of Guangzhou Huayin Medical Laboratory Center, China. As this was a retrospective study, the requirement for informed consent was waived. This study is reported following the Transparent Reporting of a Multivariable Prediction Model for Individual Prognosis or Diagnosis (TRIPOD) reporting guideline.

### Study Design and Participants

**Figure 1.**
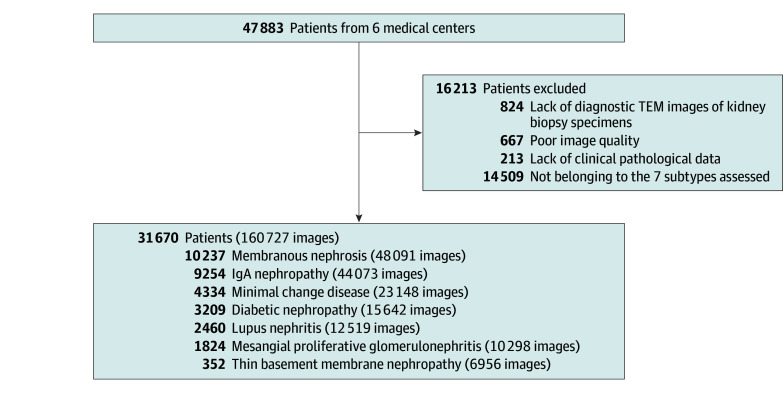
Flowchart of Patient Enrollment and Image Selection IgA indicates immunoglobin A; TEM, transmission electron microscopy.

In this retrospective multicenter diagnostic study, a total of 160 727 TEM images from 31 670 patients with chronic kidney disease were collected from 6 medical centers from January 2021 to December 2023 (Figure 1; eTable 1 in Supplement 1). Among these, 26 650 patients with glomerular disease who underwent renal biopsy from Huayin Medical Laboratory Center (Guangzhou, China) were divided into training and validation sets at an 8:2 ratio (21 320 vs 5330). Five independent external test cohorts, including Donghua Hospital (Dongguan, China; 1226 patients), Fujian Medical University Affiliated Second Hospital (Quanzhou, China; 875 patients), Gaozhou People’s Hospital (Gaozhou, China; 933 patients), Handan Central Hospital (Handan, China; 962 patients), and Xiamen University Affiliated Zhongshan Hospital (Xiamen, China; 1024 patients) were used to evaluate the generalizability and versatility of the model. Furthermore, to compare the diagnostic performance of the model, we recruited 4 pathologists and conducted a human-AI confrontation test on the 454-patient validation set. More details on study design and participants are provided in eMethods 1 and 2 in Supplement 1.

### Architecture of TEM-AID

We developed TEM-AID, a system including detection, segmentation, measurement, and classification modules that encompasses the entire TEM diagnostic workflow (eMethods 3-5 and eFigure 1 in Supplement 1). The system included 3 main parts. First, the segmentation part was responsible for accurate pixel-level segmentation of the GBM, podocytes, and EDDs. Second, the measurement part was used to accurately quantify and statistically analyze the different segmented components. Lastly, the classification part categorized the subtypes of glomerular disease based on the results of the previous 2 parts.

In the segmentation task, our proposed TEM-AID model integrates the YOLO-v8 detection model and the segment anything model (SAM),^18^ and incorporates a human-in-the-loop mechanism,^19^ enabling instance segmentation of GBMs, FPs, and EDDs. Among them, YOLO-v8 is used to generate a bounding box for each object, and SAM generates pixel-level segmentation masks based on the bounding boxes, which serves as the final result for the subsequent measurement task (eAppendix in Supplement 1). The human-in-the-loop mechanism addresses the problem of insufficient annotations through multiple cyclic iterative processes of annotation, training, prediction, and annotation.

In the measurement task, we focused on 3 aspects: measurement of GBM thickness, evaluation of podocyte fusion degree, and determination of the presence or absence of EDDs. We used the Shapely and OpenCV libraries for these measurements. First, the contour of the GBM was detected and divided into inner and outer sides. For each pixel on the inner side, its thickness value was determined by calculating the distance from this pixel to the nearest pixel on the outer side. Finally, the mean thickness of the GBM was obtained by calculating the mean of all these thickness values. The evaluation of podocyte fusion degree adopted a 4-level grading standard: less than 10%, 10% to 50%, 50% to 80%, and more than 80%, corresponding to no fusion, segmental fusion, extensive fusion, and diffuse fusion, respectively (detailed measurement methods are provided in the eAppendix in Supplement 1). For EDDs, only the presence or absence was judged. Due to the limitation of annotated data, it is currently impossible to determine the deposition sites.

In the feature fusion and selection stage, we used the feature maps output by the SAM image encoder in TEM-AID as deep features, while simultaneously integrating multiple measurement results from the measurement tasks as statistical features. Meanwhile, we combined various measurement results from the measurement task to create statistical features. These 2 types of features were concatenated, and the least absolute shrinkage and selection operator algorithm was used for feature selection. In the classification stage, we proposed a stacking classifier consisting of a meta-classifier and 4 base classifiers. The meta-classifier was a linear regression model, and the 4 base classifiers were supported vector machine, eXtreme gradient boosting, K-nearest neighbors, and light gradient boosting machine. By training the stacking classifier, we obtained the classification results for the 7 subtypes, thereby improving the accuracy of the results (eFigure 1 and eAppendix in Supplement 1).

### Statistical Analysis

To evaluate the performance of TEM-AID in the whole segmentation, measurement, and classification tasks, we used mean Dice, mean intersection over union (IOU), and mean absolute error metrics as evaluation standards. For the measurement of GBM thickness, the presence of fused FPs, and the deposition sites of EDDs, we used area under the receiver operating characteristic curve (AUC), accuracy, F1 score, precision, and recall as metrics. Finally, for subtype classification, we used accuracy and confusion matrices for evaluation. In the 2-tailed analysis, *P* < .05 was considered statistically significant. All statistical analyses were performed using Python software version 3.10 (Python Software Foundation), R software version 3.4.0 (R Project for Statistical Computing), SPSS software version 25.0 (IBM), and scikit-learn package version 0.21.3 (Scikit-learn). Data were analyzed from January to December 2024.

## Results

### Performance of TEM-AID in Predicting Glomerular Disease Subtypes

**Figure 2.**
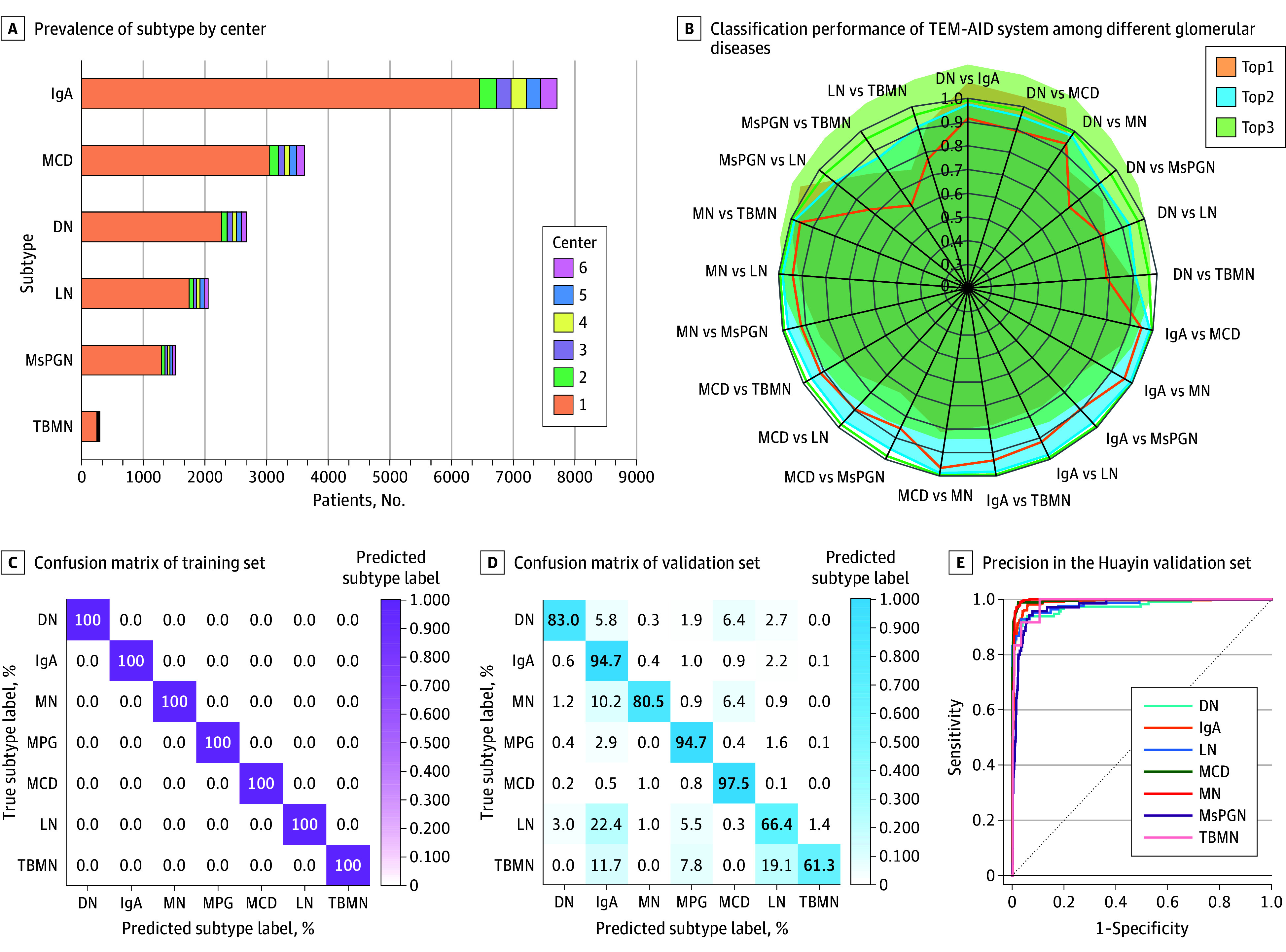
The Performance of TEM-AID System in Glomerular Disease Classification DN indicates diabetic nephropathy; IgA, immunoglobin A nephropathy; LN, lupus nephritis; MCD, minimal change disease; MN, membranous nephrosis; MsPGN, mesangial proliferative glomerulonephritis; TBMN, thin basement membrane nephropathy; TEM-AID, transmission electron microscopy image–based artificial intelligence–assisted device. Top1 indicates the prediction is correct only if the most confident class matches the true label; Top2, 1 of the 2 most confident classes; Top3, prediction is correct if the true label is 1 of the 3 most confident classes;

**Figure 3.**
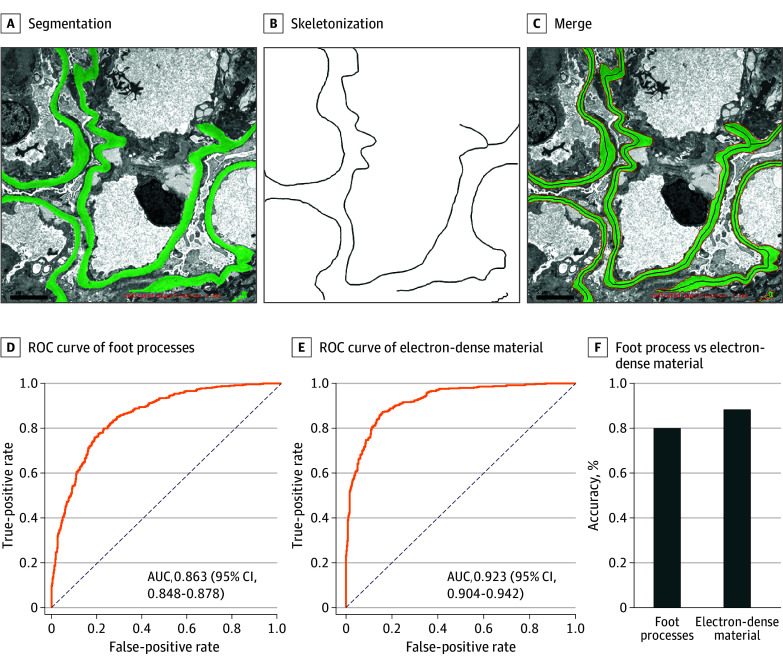
Precise Segmentation, Skeletonization, and Thickness Measurement of Glomerular Basement Membrane and Performance of TEM-AID Segmentation Foot Process and Electron-Dense Deposits AUC indicates area under the receiver operating characteristic (ROC) curve; TEM-AID, transmission electron microscopy image–based artificial intelligence–assisted device.

The TEM-AID system was trained and validated on 160 727 images from 31 670 patients (mean [SD] age, 43.2 [16.5] years; 17 372 [54.9%] male ). Figure 2B-E and eFigures 2 and 3 in Supplement 1 illustrate the performance of TEM-AID in the glomerular disease subtype classification task: it achieved near 100% accuracy on the training set, with accuracy ranging from 0.895 to 0.914 on the validation set and 5 external test sets. Specifically, in internal validation experiments, the accuracy of TEM-AID was 0.911 (95% CI, 0.904-0.918); for external test sets, the accuracies were 0.914 (95% CI, 0.898-0.930) for test set A, 0.895 (95% CI, 0.876-0.914) for test set B, 0.897 (95% CI, 0.879-0.915) for test set C, 0.895 (95% CI, 0.877-0.913) for test set D, and 0.914 (95% CI, 0.896-0.931) for test set E. Detailed subtype classification results are shown in the confusion matrices in Figure 3C-D and eFigure 2 in Supplement 1. Additionally, other performance metrics, including receiver operating characteristic curves, F1 scores, sensitivity, specificity, and precision, are provided in eTables 2 to 8 and eFigure 2 in Supplement 1 and in Figure 3E. This comprehensive evaluation highlights the reliability and robustness of TEM-AID in subtype determination across different datasets.

Given that this study addressed a multiclass system rather than a binary one, we computed and illustrated the AUC for each class. To further facilitate result assessment, we also computed macro- and micro-AUCs, which consolidate the AUCs of all subcategories into single entities. Specifically, the macro-AUCs for the validation set were 0.989 (95% CI, 0.988-0.991) in the validation set, 0.985 (95% CI, 0.978-0.992) in test A, 0.976 (95% CI, 0.966-0.986) in test B, 0.972 (95% CI, 0.961-0.983) in test C, 0.975 (95% CI, 0.965-0.985) in test D, and 0.986 (95% CI, 0.979-0.993) in test E. These comprehensive metrics provide a robust evaluation of TEM-AID’s performance across multiple classes. Detailed results are presented in Figure 3, including individual AUCs for each class, thereby offering a nuanced understanding of the model’s classification capabilities.

Additionally, decision curve analysis was used to evaluate the clinical utility of TEM-AID in identifying 7 subtypes of glomerular disease. The results indicate that TEM-AID provided a higher net benefit for patients across a range of threshold probabilities (eFigures 4-8 in Supplement 1).

### GBM Segmentation and Measurement

We performed instance detection and segmentation of the GBM using the YOLO-v8/SAM model to obtain GBM masks (eFigures 9 and 10 in Supplement 1). This approach yielded excellent performance, with a mean (SD) IOU of 0.835 (0.062) and a Dice score of 0.874 (0.023) (eFigures 1 and 11-20 and eTable 9 in Supplement 1), highlighting its precision and reliability. These results highlight the precision and reliability of TEM-AID. However, given the nonuniform thickness of the GBM, we developed a series of measurement steps using these GBM masks to accurately quantify GBM thickness. First, we extracted each polygonal segmentation region and excluded those with small areas. We then removed residual noise and small artifacts via morphological opening. For each mask’s contour, we computed its perimeter, area, and compactness and smoothed the contour using polygonal approximation (eFigure 1 in Supplement 1). Next, we skeletonized the filled contours to obtain a single pixel–width centerline, which represents the GBM’s main structure. Using this skeleton, we calculated the mean thickness of each contour by dividing its area by the skeleton length (Figure 2A; eFigure 21 in Supplement 1). To ensure accuracy, we computed the skeleton’s curvature and number of branch points, normalized these values, and used their standard deviations to assess the skeleton’s smoothness and complexity—this helped filter out false positive detections (Figure 2A; eFigure 21 in Supplement 1). Finally, we selected contours meeting criteria based on compactness, fill rate, normalized curvature, and normalized branch points. This process enabled accurate measurement of GBM thickness, providing reliable data for the diagnosis and subtyping of glomerular diseases (eFigure 1 in Supplement 1).

### Segmentation and Classification of FP and EDDs

In addition to GBM thickness, EDD deposition and FP fusion status are also key indicators for pathologists in diagnosing different glomerular disease subtypes. We obtained instance segmentation masks of EDD and FP using the YOLO-v8/SAM model. For FP, we derived the FP density based on the relationship between the number of detected FPs and the length of the basement membrane and used this to classify patients into those with diffuse FP fusion and those without. In actual testing, our method achieved an accuracy of 0.801 (95% CI, 0.786-0.816) and an AUC of 0.863 (95% CI, 0.848-0.878) (Figure 3D and F). For EDD, pathologists typically diagnose both the presence of EDD and the locations of deposition. However, since our data lack annotations of deposition locations, we only diagnose the presence or absence of EDD. Here, we classified patients into 2 categories: those with EDD and those without. For this task, we achieved an accuracy of 0.89X (95% CI, 0.868-0.906) and an AUC of 0.92X (95% CI, 0.904-0.942) (Figure 3E and F).

### Human-AI Test

**Figure 4.**
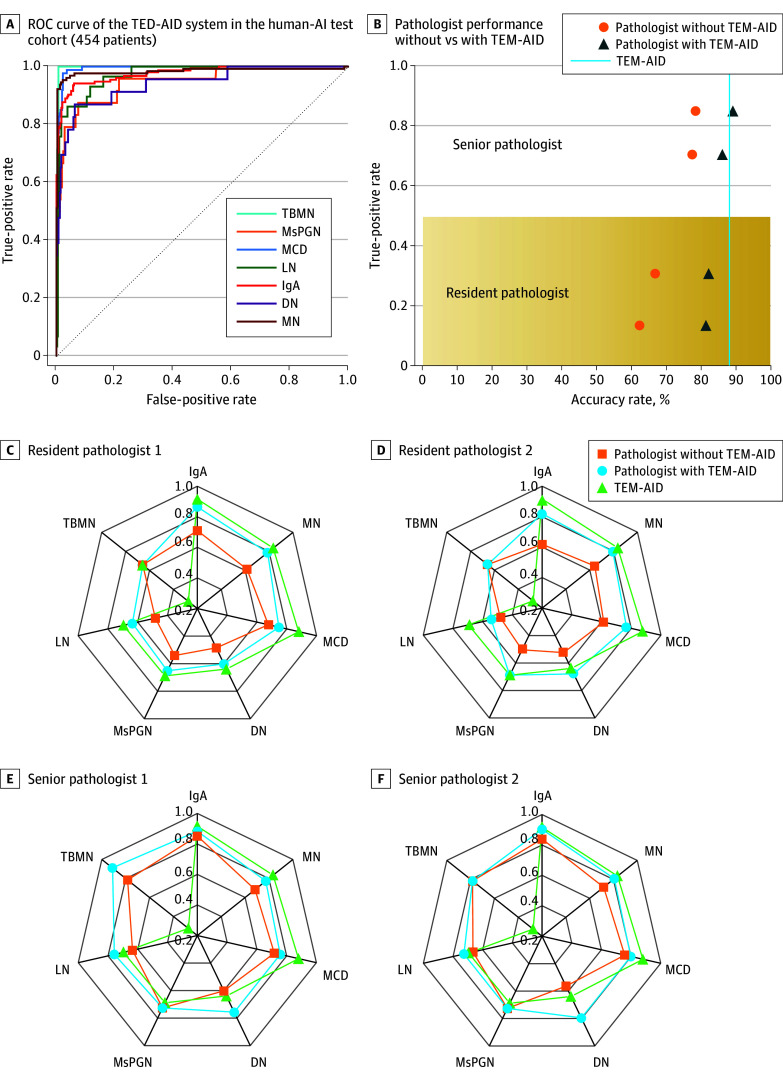
The Results of Human–Artificial Intelligence Test AI indicates artificial intelligence; AUC, area under the receiver operating characteristic (ROC) curve; DN, diabetic nephropathy (AUC, 0.941 [95% CI, 0.915-0.967]); IgA, immunoglobin A nephropathy (AUC, 0.977 [95% CI, 0.951-1.000]); LN, lupus nephritis (AUC, 0.971 [95% CI, 0.945-0.997]); MCD, minimal change disease (AUC, 0.993 [95% CI, 0.967-1.000]); MN, membranous nephrosis (AUC, 0.984 [95% CI, 0.958-1.000]); MsPGN, mesangial proliferative glomerulonephritis (AUC, 0.947 [95% CI, 0.921-0.973]); TBMN, thin basement membrane nephropathy (AUC, 0.998 [95% CI, 0.972-1.000]); TEM-AID, transmission electron microscopy image–based AI-assisted device.

In the human-AI test experiments, a total of 454 patients were selected for diagnosis. The TEM-AID system significantly surpassed the mean accuracy level of 0.724 (95% CI, 0.698-0.750) of clinicians, with an accuracy of 0.886 (95% CI, 0.859-0.912) and an AUC of 0.963 (95% CI, 0.937-0.989) (Figure 4A; eTables 10 and 11 and eFigure 22 in Supplement 1). Four pathologists were invited to participate in this experiment, and their accuracy rates were 0.674, 0.639, 0.784, and 0.800. However, after the introduction of TEM-AID, their accuracy rates were significantly improved, reaching 0.822, 0.813, 0.852, and 0.877, respectively, with a mean (SD) increase of 11.7% (5.2%) (Figure 4B-F), showcasing the practical value of AI in assisting clinical decision-making.

## Discussion

This diagnostic study describes the TEM-AID system, which enables automated, high-precision assessment of 7 major glomerular disease types in TEM images by integrating detection, segmentation, measurement, and classification functions. Renal electron microscopy diagnosis has garnered growing interest among clinicians and researchers.^18,22,23^ To our knowledge, TEM-AID is the first clinically applicable AI system for the diagnosis and quantitative analysis of glomerular diseases. It provided reliable evaluation of critical ultrastructural features, including the GBM, EDDs, and podocyte FPs. Multicenter validation across 5 Chinese medical institutions confirmed its generalizability, with clinical-grade accuracy, sensitivity, and specificity. Compared with traditional manual analysis relying on experienced pathologists, the TEM-AID system demonstrated significant advantages: not only did it excel in diagnostic accuracy, more importantly, it was associated with substantially improved analysis efficiency and objectivity, effectively addressing the issues of subjective variability and efficiency bottlenecks inherent in conventional manual analysis. Therefore, the TEM-AID system represents a groundbreaking tool that provides robust support for the standardized and quantitative assessment of glomerular diseases. It holds promise for assisting clinicians in leveraging TEM images more efficiently and accurately for diagnostic decision-making, thereby advancing the overall level of related diagnosis and treatment.

The proposed TEM-AID model innovatively combines SAM^24^ with a human-AI collaboration mechanism^25^ to achieve precise TEM image segmentation. By adapting SAM’s natural image capabilities to medical imaging, we overcome domain shift challenges. The iterative human-AI collaboration significantly reduced annotation burden while improving accuracy—initial model predictions were refined by experts to expand training data. This approach demonstrated superior performance compared with conventional methods, offering an efficient solution for clinical TEM analysis. The improvement was especially notable in cases where the clinicians encountered ambiguous or complex diagnoses, as TEM-AID provided valuable insights and highlighted areas of uncertainty. Furthermore, the variability in clinician performance was reduced with the use of TEM-AID, indicating that the system helped standardize and enhance diagnostic accuracy across different individuals. Future work will extend to 3-dimensional segmentation and multimodal integration.

In the subtype classification task, we simultaneously used deep features obtained from the segmentation task and precise statistical features obtained from the measurement task. Both types of features are crucial for the final classification performance. On the one hand, although deep features lack direct interpretability, they can capture subtle structures that are difficult for human physicians to detect, making the model more sensitive to variations in different images. On the other hand, statistical features enhance the robustness and interpretability of the stage-based classification, facilitating our adoption and acceptance of the classification results. Furthermore, stacking-based classifiers have demonstrated performance advantages over single classifiers, showcasing the effectiveness of the combined use of multiple classifiers in improving the overall performance.

Although a stack ensemble significantly improves the classification accuracy compared with simple models, it essentially increases the model complexity and reduces the transparency. This black box characteristic poses challenges to clinical applications, because understanding the principles behind the diagnosis is crucial for pathologists’ trust, error debugging, and regulatory adherence. Improving the interpretability of the classification stage of the TEM-AID system is a key direction for the future. Potential strategies include adopting inherently interpretable models (eg, decision trees, explainable boosting machine algormthms^26^), applying post hoc techniques (eg, Shapley additive explanations,^27^ local interpretable model-agnostic explanations^28^), developing integrated visualization tools to overlay the prediction results with key segmentation structures (eg, through gradient-weighted class activation mapping^29^) and statistical measurement results, and designing a human-machine collaborative workflow that enables TEM-AID to provide interpretable evidence (visualization, key indicators, confidence) while presenting the prediction results so that pathologists can review and override them efficiently. Achieving a better balance between high performance and interpretability is essential for enhancing clinical confidence and smoothly integrating into the diagnostic workflow.

### Limitations

This study has several limitations. First, our proposed TEM-AID system was built on multicenter retrospective data, and although it achieved excellent performance in 5 external validation sets and a human-AI test set, further validation of the system’s validity and performance in prospective cohorts is needed in the future. The external validation was conducted exclusively in Chinese centers, which might limit the generalizability of our model to other regions. Second, TEM-AID is still only intended for diagnosis and analysis of glomerular disease and has not been applied and validated in a wider range of kidney diseases. Third, the 4 components of TEM-AID are independent of each other, which reduces interactions but increases the complexity of the system and reduces fault tolerance, so a more unified foundation model could be designed to realize all tasks in 1 model.

## Conclusions

This multicenter diagnostic study, TEM-AID precisely quantified glomerular ultrastructures and determined glomerular disease subtypes from TEM images, significantly enhancing diagnostic efficiency and accuracy. These findings suggest that this system can provide quantitative evaluation tools to support clinical pathologists in diagnostic workflows, demonstrating robust multicenter performance.

## Notes

Supplement 1. eMethods 1. Study design and participants eMethods 2. Inclusion and exclusion criteria, and data sources eMethods 3. Details of the YOLOv8-SAM model for object detection and segmentation in TEM image eMethods 4. Architecture of the TEM-AID system eMethods 5. Training process of the TEM-AID system eTable 1. Baseline characteristics among six centers eTable 2. AUC values of TEM-AID in classifying seven subtypes of glomerulonephritis among validation and test datasets eTable 3. Prediction performance of TEM-AID in the validation dataset eTable 4. Prediction performance of TEM-AID in the test set A eTable 5. Prediction performance of TEM-AID in the test set B eTable 6. Prediction performance of TEM-AID in the test set C eTable 7. Prediction performance of TEM-AID in the test set D eTable 8. Prediction performance of TEM-AID in the test set E eTable 9. Performance of glomerular basement membrane segmentation eTable 10. Professional training and working experiences of pathology experts eTable 11. Human-AI test results eFigure 1. Workflow of the proposed TEM-AID and study design eFigure 2. Confusion Matrix and the ROC analysis of five external test datasets eFigure 3. Eight-class confusion matrices for TEM-AID performance on Huayin datasets eFigure 4. DCA Curve of TEM-AID over seven subtypes of glomerulonephritis in the validation set eFigure 5. DCA Curve of TEM-AID over seven subtypes of glomerulonephritis in the test set A eFigure 6. DCA Curve of TEM-AID over seven subtypes of glomerulonephritis in the test set B eFigure 7. DCA Curve of TEM-AID over seven subtypes of glomerulonephritis in the test set C eFigure 8. DCA Curve of TEM-AID over seven subtypes of glomerulonephritis in the test set E eFigure 9. Architecture of YOLOv8-SAM eFigure 10. Architecture of TEM-AID eFigure 11. Box Precision-Recall eFigure 12. Box Recall-Confidence Curve eFigure 13. Labels Distribution eFigure 14. Mask Precision-Recall Curve eFigure 15. Mask Precision-Confidence Curve eFigure 16. Mask F1-Confidence Curve eFigure 17. Mask Recall-Confidence Curve eFigure 18. Training Results eFigure 19. Box F1-Confidence Curve eFigure 20. Box Precision-Confidence eFigure 21. TEM-AID segmentation of glomerular basement membranes, electron-dense material and foot processes eFigure 22. The results of human-AI test eAppendix. Detailed Explanation of TEM-AID system

Supplement 2. Data Sharing Statement
