# Supplementary material for: AI-Based System for Analysis of Electron Microscope Images in Glomerular Disease
Source: JAMA Netw Open. 2025 Oct 7;8(10):e2534985. doi: 10.1001/jamanetworkopen.2025.34985 (PMC12505174; doi:10.1001/jamanetworkopen.2025.34985)
Supplement: Supplement 1. — eMethods 1. Study design and participants eMethods 2. Inclusion and exclusion criteria, and data sources eMethods 3. Details of the YOLOv8-SAM model for object detection and segmentation in TEM image eMethods 4. Architecture of the TEM-AID system eMethods 5. Training process of the TEM-AID system eTable 1. Baseline characteristics among six centers eTable 2. AUC values of TEM-AID in classifying seven subtypes of glomerulonephritis among validation and test datasets eTable 3. Prediction performance of TEM-AID in the validation dataset eTable 4. Prediction performance of TEM-AID in the test set A eTable 5. Prediction performance of TEM-AID in the test set B eTable 6. Prediction performance of TEM-AID in the test set C eTable 7. Prediction performance of TEM-AID in the test set D eTable 8. Prediction performance of TEM-AID in the test set E eTable 9. Performance of glomerular basement membrane segmentation eTable 10. Professional training and working experiences of pathology experts eTable 11. Human-AI test results eFigure 1. Workflow of the proposed TEM-AID and study design eFigure 2. Confusion Matrix and the ROC analysis of five external test datasets eFigure 3. Eight-class confusion matrices for TEM-AID performance on Huayin datasets eFigure 4. DCA Curve of TEM-AID over seven subtypes of glomerulonephritis in the validation set eFigure 5. DCA Curve of TEM-AID over seven subtypes of glomerulonephritis in the test set A eFigure 6. DCA Curve of TEM-AID over seven subtypes of glomerulonephritis in the test set B eFigure 7. DCA Curve of TEM-AID over seven subtypes of glomerulonephritis in the test set C eFigure 8. DCA Curve of TEM-AID over seven subtypes of glomerulonephritis in the test set E eFigure 9. Architecture of YOLOv8-SAM eFigure 10. Architecture of TEM-AID eFigure 11. Box Precision-Recall eFigure 12. Box Recall-Confidence Curve eFigure 13. Labels Distribution eFigure 14. Mask Precision-Recall Curve eFigure 15. Mask Precision-Confidence Curve eFigure 16. Mask F1 [file jamanetwopen-e2534985-s001.pdf]

Supplemental Online Content

Ma P, Li J, Zhang Z, et al. AI-based system for analysis of electron microscope images in glomerular disease. *JAMA Netw Open*. 2025;8(10):e2534985.
doi:10.1001/jamanetworkopen.2025.34985

eMethods 1. Study design and participants
eMethods 2. Inclusion and exclusion criteria, and data sources
eMethods 3. Details of the YOLOv8-SAM model for object detection and segmentation in TEM image
eMethods 4. Architecture of the TEM-AID system
eMethods 5. Training process of the TEM-AID system
eTable 1. Baseline characteristics among six centers
eTable 2. AUC values of TEM-AID in classifying seven subtypes of glomerulonephritis among validation and test datasets
eTable 3. Prediction performance of TEM-AID in the validation dataset
eTable 4. Prediction performance of TEM-AID in the test set A
eTable 5. Prediction performance of TEM-AID in the test set B
eTable 6. Prediction performance of TEM-AID in the test set C
eTable 7. Prediction performance of TEM-AID in the test set D
eTable 8. Prediction performance of TEM-AID in the test set E
eTable 9. Performance of glomerular basement membrane segmentation
eTable 10. Professional training and working experiences of pathology experts
eTable 11. Human-AI test results
eFigure 1. Workflow of the proposed TEM-AID and study design
eFigure 2. Confusion Matrix and the ROC analysis of five external test datasets
eFigure 3. Eight-class confusion matrices for TEM-AID performance on Huayin datasets
eFigure 4. DCA Curve of TEM-AID over seven subtypes of glomerulonephritis in the validation set
eFigure 5. DCA Curve of TEM-AID over seven subtypes of glomerulonephritis in the test set A
eFigure 6. DCA Curve of TEM-AID over seven subtypes of glomerulonephritis in the test set B
eFigure 7. DCA Curve of TEM-AID over seven subtypes of glomerulonephritis in the test set C
eFigure 8. DCA Curve of TEM-AID over seven subtypes of glomerulonephritis in the test set E
eFigure 9. Architecture of YOLOv8-SAM
eFigure 10. Architecture of TEM-AID
eFigure 11. Box Precision-Recall
eFigure 12. Box Recall-Confidence Curve
eFigure 13. Labels Distribution
eFigure 14. Mask Precision-Recall Curve
eFigure 15. Mask Precision-Confidence Curve
eFigure 16. Mask F1-Confidence Curve
eFigure 17. Mask Recall-Confidence Curve
eFigure 18. Training Results
eFigure 19. Box F1-Confidence Curve
eFigure 20. Box Precision-Confidence
eFigure 21. TEM-AID segmentation of glomerular basement membranes, electron-dense material and foot processes
eFigure 22. The results of human-AI test
eAppendix. Detailed Explanation of TEM-AID system

This supplemental material has been provided by the authors to give readers additional information about their work.

© 2025 Ma P et al. *JAMA Network Open*.

1. emethods

eMethod 1. Study design and participants.

The inclusion criteria for this study were as follows: (1) patients diagnosed with glomerular diseases confirmed by light microscopy, immunofluorescence examination, and TEM examination, reviewed by two experienced pathologists, with cases having a definite diagnosis included; (2) disease selection: including seven common glomerular diseases such as IgA nephropathy, membranous nephropathy, thin basement membrane nephropathy, minimal change disease, mesangial proliferative GN, lupus nephritis, and diabetic nephropathy; (3) image selection: TEM images with high-resolution and containing the glomerular region. Exclusion criteria for this study were as follows: (1) Lack of diagnostic TEM images of renal biopsy specimens or poor image quality; (2) Lack of complete clinical or pathological data. For each patient, there were usually multiple TEM images taken at magnifications ranging from 1000 to 6000 times. Images at lower magnifications allow for the observation of the overall structure of multiple or larger glomeruli, but the various ultrastructure may not be clear enough. Higher magnifications can provide a more detailed and clear structure of the glomerulus but may have limited content and scope.

As shown in Fig.1 and Supplementary eTable 11, we collected patients with GD from 6 centers in China from January 2021 to December 2023, a total of 31,670 cases, including 7 subtypes, namely: diabetic nephropathy (DN, 3,209 cases, 10.1%), IgA nephropathy (IgA, 9,254 cases, 29.2%), membranous nephroitis (MN, 10,237 cases, 32.3%), mesangial proliferative glomerulonephritis (MsPGN, 1,824 cases, 5.7%), minimal change disease (MCD, 4,334 cases, 13.7%), lupus nephritis (LN, 2,460 cases, 7.8%) and thin basement membrane nephropathy (TBMN, 352 cases, 1.1%) . All data were divided into a training set, an internal validation set and 5 external testing sets (Supplementary eTable 11). However, the data imbalance, with TBMN accounting for only 1.1%, partially presents challenges for model development and validation. Among them, the data from Center 1-Guangzhou Huayin Medical Laboratory Center had a total of 26,650 (84.1%) cases, which were divided into a training set and an internal validation set according to the principle of 8:2 (21,320 cases vs 5,330 cases). Center 2, Dongguan Tungwah Hospital with a total of 1,226 (3.9%) cases, was defined as test set A. Center 3, Fujian Medical University 2nd Affiliated Hospital with a total of 875 (2.8%) cases, was defined as test set B. Center 4, Gaozhou People's Hospital with a total of 933 (3.0%) cases, was defined as test set C. Center 5, Handan Central Hospital with a total of 962 (3.0%) cases, was defined as test Set D. Center 6, Zhongshan Hospital Xiamen University with a total of 1,024 (3.2%) cases, was defined as test set E. In addition, Alport Syndrome accounting for only 0.1%, Alport syndrome is a relatively rare condition, with an incidence of approximately 1 in 50,000 live births. Given the focus of our current study on more prevalent glomerular diseases, the low incidence of Alport syndrome presented a challenge in terms of data availability and sample size. However, we still attempted to incorporate the Alport category to validate its effectiveness. We identified a total of 42 Alport cases from the Huayin Center, comprising 524 TEM images, which were randomly divided into training and validation sets at an 8:2 ratio. This adjustment expanded our overall categories from 7 to 8. Among all 31,712 patients, the proportions of males and females were 54.9% and 45.1% respectively. In terms of age, 19,05 cases (6.0%) were younger than 18 years old, 10,551 cases

(33.3%) were 18-39 years old, 13,742 cases (43.4%) were 40-59 years old, and 5,381 cases (17.0%) were 60-79 years old. 91 cases (0.3%) were older than 80 years old. Each patient had 2 to 3 4000× TEM images (Table 1). We excluded images that were blurry, irregularly photographed, too bright or too dark, or damaged. There were no statistically significant differences in baseline characteristics between patients across all centers.

eMethod 2. Inclusion and exclusion criteria, and data sources.

All the patients in this study satisfied the following inclusion criteria: (i) patients diagnosed with glomerular diseases confirmed by light microscopy, immunofluorescence examination, and TEM examination, reviewed by two experienced pathologists, with only cases having a definite diagnosis included; (ii) disease selection: including seven common glomerular diseases such as IgA nephropathy, membranous nephropathy, thin basement membrane nephropathy, minimal change disease, mesangial proliferative glomerulonephritis, lupus nephritis, and diabetic nephropathy; (iii) image selection: TEM images with a magnification of 4000 times (format: JPG, pixel: 2048*2048). Exclusion criteria for this study were as follows: (i) Lack of diagnostic TEM images of renal biopsy specimens or poor image quality; (ii) Lack of complete clinical or pathological data.

eMethod 3. Details of the YOLOv8-SAM model for object detection and segmentation in TEM image.

To detect and segment the basement membranes, foot processes and electron dense matters accurately from TEM images, we use YOLOv8 as the objection detection component and SAM as object segmentation component. YOLOv8 is the newest state-of-the-art YOLO model that can be used for object detection, image classification, and instance segmentation tasks. YOLOv8 was developed by Ultralytics, who also created the influential and industry-defining YOLOv5 model. YOLOv8 has a high rate of accuracy measured by COCO and Roboflow 100. YOLOv8 comes with a lot of developer-convenience features, from an easy-to-use CLI to a well-structured Python package. The architecture of YOLOv8-SAM is shown in Supplementary Figure 1.

© 2025 Ma P et al. *JAMA Network Open.*

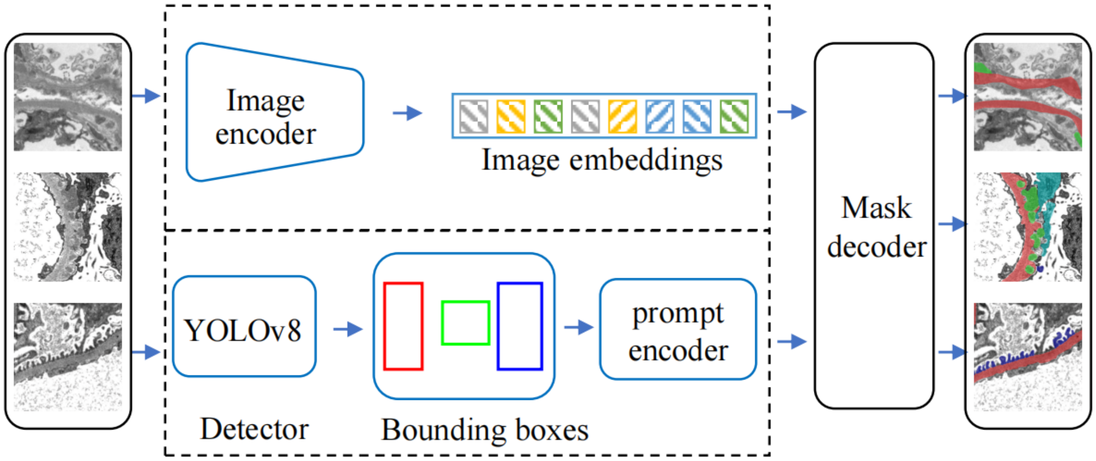

Supplementary Figure 1. Architecture of YOLOv8-SAM

YOLOv8 is an anchor-free model. Anchor boxes were a notoriously tricky part of earlier YOLO models, since they may represent the distribution of the target benchmark's boxes but not the distribution of the custom dataset. Anchor free detection reduces the number of box predictions, which speeds up Non-Maximum Suppression (NMS), a complicated post processing step that sifts through candidate detections after inference. In the neck, features are concatenated directly without forcing the same channel dimensions. This reduces the parameters count and the overall size of the tensors.

YOLOv8 research was primarily motivated by empirical evaluation on the COCO benchmark. As each piece of the network and training routine are tweaked, new experiments are run to validate the changes effect on COCO modeling.

© 2025 Ma P et al. *JAMA Network Open.*

eMethod 4. Architecture of the TEM-AID system.

The TEM-AID system consists of 4 modules: detection module, segmentation module, calculation module and classification module. In the detection module, we use the yolov8 algorithm to detect basement membrane, electron dense matters, and foot processes. In the segmentation module, we use the SAM algorithm to accurately segment the detected different parts and obtain their precise edges. In the calculation module, we use packages such as OpenCV and shapely to assist us in numerical calculations. Finally, we used an stacked model for classification to obtain the precise subtypes of glomerulonephritis, as shown in Supplementary Figure 2.

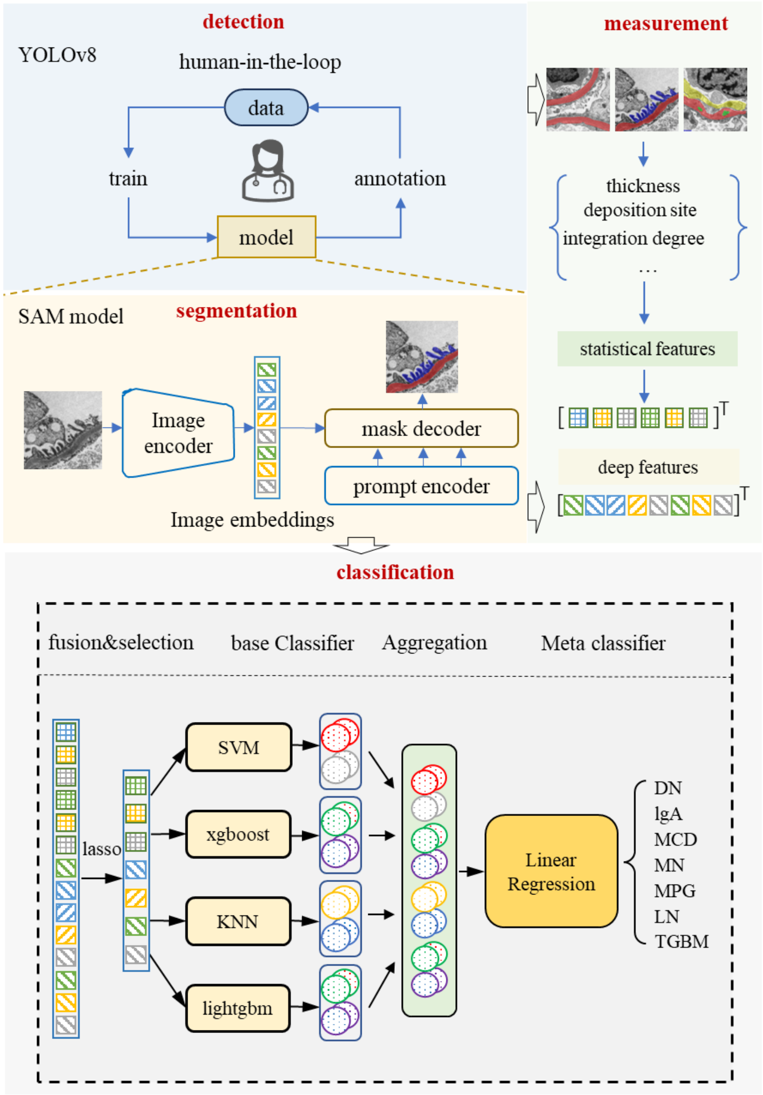

Supplementary Figure 2. Architecture of TEM-AID

© 2025 Ma P et al. *JAMA Network Open*.

eMethod 5. Training process of the TEM-AID system.

Model training aims at optimizing parameters of the TEM-AID to build the relationship between TEM image and the subtypes of glomerulonephritis. The model training is an iterative process, which optimizes the model in the training set at each iteration until the model achieves the best predictive performance in the validation set. At each iteration, we used the AUC loss as the loss function. If the loss function did not reach a minimum in the validation set, we used the stochastic gradient descent (SGD) algorithm (batch size = 24 and learning rate = 5×10-3) to update the parameters of the TEM-AID and minimize the loss function. To avoid overfitting, data augmentation was used, including random rotation in the range of [-5, 5] degrees and random shift in the range of [-5%, 5%] voxels.

2. eTables

eTable 1. Baseline characteristics among six centers

	Center 1 (n=26650)	Center 2 (n=1226)	Center 3 (n=875)	Center 4 (n=933)	Center 5 (n=962)	Center 6 (n=1024)
Age, n (%)						
<18	1617 (6.0%)	75 (6.1%)	44 (5.0%)	51 (5.5%)	54 (5.6%)	64 (6.3%)
18-39	8932 (33.5%)	393 (32.0%)	290 (33.1%)	280 (30.0%)	320 (33.3%)	336 (32.8%)
40-59	11539 (43.3%)	530 (43.2%)	395 (45.1%)	419 (44.9%)	404 (42.0%)	455 (44.4%)
60-79	4490 (16.9%)	223 (18.2%)	142 (16.2%)	181 (19.4%)	179 (18.6%)	166 (16.2%)
≥80	72 (0.3%)	5 (0.4%)	4 (0.5%)	2 (0.2%)	5 (0.5%)	3 (0.3%)
Sex, n (%)						
Male	14643 (54.9%)	684 (55.8%)	490 (56.0%)	477 (51.1%)	526 (54.7%)	552 (53.9%)
Female	12007 (45.1%)	542 (44.2%)	385 (44.0%)	456 (48.9%)	436 (45.3%)	472 (46.1%)
Disease, n (%)						
DN	2719 (10.2%)	115 (9.4%)	96 (11.0%)	84 (9.00%)	101 (10.5%)	94 (9.2%)
IgA	7749 (29.1%)	328 (26.8%)	278 (31.8%)	305 (32.7%)	278 (28.9%)	316 (30.9%)
LN	2095 (7.9%)	83 (6.8%)	59 (6.7%)	66 (7.1%)	83 (8.6%)	74 (7.2%)
MCD	3650 (13.7%)	185 (15.1%)	106 (12.1%)	107 (11.5%)	132 (13.7%)	154 (15.0%)
MN	8585 (32.2%)	433 (35.3%)	282 (32.2%)	313 (33.5%)	306 (31.8%)	318 (31.1%)
MsPGN	1555 (5.8%)	70 (5.7%)	48 (5.5%)	48 (5.1%)	51 (5.3%)	52 (5.1%)
TBMN	297 (1.1%)	12 (1.0%)	6 (0.7%)	10 (1.1%)	11 (1.1%)	16 (1.6%)

DN = Diabetic Nephropathy. IgA = IgA Nephropathy. MCD = Minimal Change Disease. MN = Membranous Nephrosis. MsPGN = Mesangial Proliferative Glomerulonephritis. LN = Lupus Nephritis. TBMN= Thin Basement Membrane Nephropathy.

2.2 eTable 2. AUC values of TEM-AID in classifying seven subtypes of glomerulonephritis among validation and test datasets

	Validation	Test A	Test B	Test C	Test D	Test E
DN	0.982(0.978,0.985)	0.976(0.958,0.994)	0.947(0.919,0.975)	0.926(0.884,0.968)	0.964(0.940,0.988)	0.984(0.970,0.997)
IGA	0.988(0.987,0.990)	0.989(0.983,0.995)	0.99(0.986,0.995)	0.991(0.987,0.996)	0.986(0.978,0.993)	0.991(0.986,0.997)
LN	0.988(0.985,0.990)	0.983(0.968,0.997)	0.968(0.932,1.000)	0.964(0.932,0.996)	0.986(0.972,1.000)	0.983(0.966,1.000)
MCD	0.996(0.995,0.996)	0.996(0.993,0.999)	0.997(0.993,1.000)	0.989(0.979,0.998)	0.991(0.982,1.000)	0.996(0.993,0.999)
MN	0.996(0.995,0.997)	0.995(0.992,0.999)	0.990(0.983,0.996)	0.994(0.990,0.998)	0.990(0.984,0.996)	0.994(0.989,0.998)
MsPGN	0.976(0.973,0.979)	0.974(0.960,0.988)	0.954(0.930,0.977)	0.944(0.918,0.970)	0.947(0.911,0.983)	0.973(0.960,0.987)
TBMN	0.994(0.992,0.997)	0.986(0.968,1.000)	0.990(0.982,0.999)	0.996(0.992,1.000)	0.964(0.938,0.990)	0.984(0.965,1.000)
Marco	0.989(0.988,0.991)	0.985(0.978,0.992)	0.976(0.966,0.986)	0.972(0.961,0.983)	0.975(0.965,0.985)	0.986(0.979,0.993)
Micro	0.992(0.991,0.993)	0.992(0.987,0.997)	0.987(0.980,0.991)	0.985(0.977,0.993)	0.986(0.979,0.993)	0.992(0.987,0.998)

DN = Diabetic Nephropathy. IgA = IgA Nephropathy. MCD = Minimal Change Disease. MN = Membranous Nephrosis. MsPGN = Mesangial Proliferative Glomerulonephritis. LN = Lupus Nephritis. TBMN= Thin Basement Membrane Nephropathy. TEM-AID = a novel TEM images based artificial intelligent diagnostic and analysis system.

2.3 eTable 3. Prediction performance of TEM-AID in the validation dataset

	AUC (95%CI)	SEN (95%CI)	SPE (95%CI)	F1_score (95%CI)	Precision (95%CI)
DN	0.982(0.978,0.985)	0.83(0.812,0.847)	0.994(0.993,0.995)	0.88(0.868,0.891)	0.936(0.923,0.948)
IGA	0.988(0.987,0.990)	0.947(0.941,0.953)	0.951(0.947,0.955)	0.916(0.911,0.921)	0.887(0.879,0.896)
LN	0.988(0.985,0.990)	0.805(0.784,0.826)	0.994(0.993,0.996)	0.861(0.847,0.874)	0.926(0.91,0.94)
MCD	0.996(0.995,0.996)	0.947(0.938,0.956)	0.986(0.984,0.987)	0.931(0.924,0.938)	0.916(0.905,0.927)
MN	0.996(0.995,0.997)	0.975(0.971,0.979)	0.978(0.976,0.981)	0.964(0.961,0.968)	0.954(0.948,0.959)
MsPGN	0.976(0.973,0.979)	0.664(0.636,0.692)	0.984(0.982,0.986)	0.697(0.676,0.716)	0.733(0.704,0.76)
TBMN	0.994(0.992,0.997)	0.613(0.55,0.676)	0.999(0.998,0.999)	0.714(0.667,0.758)	0.855(0.791,0.905)

DN = Diabetic Nephropathy. IgA = IgA Nephropathy. MCD = Minimal Change Disease. MN = Membranous Nephrosis. MsPGN = Mesangial Proliferative Glomerulonephritis. LN = Lupus Nephritis. TBMN= Thin Basement Membrane Nephropathy. TEM-AID = a novel TEM images based artificial intelligent diagnostic and analysis system.

© 2025 Ma P et al. *JAMA Network Open.*

2.4 eTable 4. Prediction performance of TEM-AID in the test set A.

	AUC (95%CI)	SEN (95%CI)	SPE (95%CI)	F1_score (95%CI)	Precision (95%CI)
DN	0.976(0.958,0.994)	0.816(0.745,0.887)	0.995(0.992,0.999)	0.877(0.825,0.918)	0.949(0.885,0.983)
IGA	0.989(0.983,0.995)	0.966(0.947,0.986)	0.952(0.938,0.966)	0.922(0.899,0.94)	0.881(0.842,0.912)
LN	0.983(0.968,0.997)	0.807(0.722,0.892)	0.997(0.994,1)	0.876(0.813,0.924)	0.957(0.88,0.991)
MCD	0.996(0.993,0.999)	0.968(0.942,0.993)	0.979(0.97,0.988)	0.927(0.897,0.951)	0.891(0.839,0.93)
MN	0.995(0.992,0.999)	0.977(0.963,0.991)	0.98(0.97,0.99)	0.97(0.957,0.98)	0.963(0.941,0.979)
MsPGN	0.974(0.96,0.988)	0.529(0.412,0.646)	0.988(0.982,0.994)	0.612(0.519,0.699)	0.725(0.583,0.841)
TBMN	0.986(0.968,1)	0.333(0.067,0.6)	0.998(0.996,1)	0.444(0.215,0.692)	0.667(0.223,0.957)

DN = Diabetic Nephropathy. IgA = IgA Nephropathy. MCD = Minimal Change Disease. MN = Membranous Nephrosis. MsPGN = Mesangial Proliferative Glomerulonephritis. LN = Lupus Nephritis. TBMN= Thin Basement Membrane Nephropathy. TEM-AID = a novel TEM images based artificial intelligent diagnostic and analysis system.

2.5 eTable 5. Prediction performance of TEM-AID in the test set B.

	AUC (95%CI)	SEN (95%CI)	SPE (95%CI)	F1_score (95%CI)	Precision (95%CI)
DN	0.947(0.919,0.975)	0.719(0.629,0.809)	0.994(0.988,0.999)	0.812(0.745,0.868)	0.932(0.849,0.978)
IGA	0.99(0.986,0.995)	0.957(0.933,0.981)	0.945(0.926,0.963)	0.922(0.897,0.942)	0.889(0.848,0.923)
LN	0.968(0.932,1)	0.831(0.735,0.926)	0.991(0.985,0.998)	0.852(0.774,0.911)	0.875(0.759,0.948)
MCD	0.997(0.993,1)	0.962(0.925,0.999)	0.99(0.982,0.997)	0.944(0.904,0.971)	0.927(0.86,0.968)
MN	0.99(0.983,0.996)	0.968(0.948,0.989)	0.971(0.958,0.985)	0.955(0.934,0.97)	0.941(0.908,0.965)
MsPGN	0.954(0.93,0.977)	0.479(0.338,0.62)	0.977(0.967,0.987)	0.511(0.403,0.618)	0.548(0.387,0.702)
TBMN	0.99(0.982,0.999)	0.167(-0.132,0.465)	0.997(0.993,1)	0.2(0.025,0.556)	0.25(0.006,0.806)

DN = Diabetic Nephropathy. IgA = IgA Nephropathy. MCD = Minimal Change Disease. MN = Membranous Nephrosis. MsPGN = Mesangial Proliferative Glomerulonephritis. LN = Lupus Nephritis. TBMN= Thin Basement Membrane Nephropathy. TEM-AID = a novel TEM images based artificial intelligent diagnostic and analysis system.

© 2025 Ma P et al. *JAMA Network Open.*

2.6 eTable 6. Prediction performance of TEM-AID in the test set C.

	AUC (95%CI)	SEN (95%CI)	SPE (95%CI)	F1_score (95%CI)	Precision (95%CI)
DN	0.926(0.884,0.968)	0.679(0.579,0.778)	0.991(0.984,0.997)	0.765(0.689,0.831)	0.877(0.772,0.945)
IGA	0.991(0.987,0.996)	0.98(0.965,0.996)	0.933(0.914,0.953)	0.925(0.902,0.945)	0.876(0.837,0.91)
LN	0.964(0.932,0.996)	0.742(0.637,0.848)	0.997(0.993,1)	0.831(0.75,0.893)	0.942(0.841,0.988)
MCD	0.989(0.979,0.998)	0.972(0.941,1)	0.982(0.973,0.991)	0.92(0.877,0.952)	0.874(0.801,0.928)
MN	0.994(0.99,0.998)	0.962(0.94,0.983)	0.976(0.964,0.988)	0.957(0.938,0.972)	0.953(0.923,0.973)
MsPGN	0.944(0.918,0.97)	0.5(0.359,0.641)	0.985(0.977,0.993)	0.565(0.453,0.672)	0.649(0.475,0.798)
TBMN	0.996(0.992,1)	0.3(0.016,0.584)	1(1,1)	0.462(0.192,0.749)	1(0.292,1)

DN = Diabetic Nephropathy. IgA = IgA Nephropathy. MCD = Minimal Change Disease. MN = Membranous Nephrosis. MsPGN = Mesangial Proliferative Glomerulonephritis. LN = Lupus Nephritis. TBMN= Thin Basement Membrane Nephropathy. TEM-AID = a novel TEM images based artificial intelligent diagnostic and analysis system.

2.7 eTable 7. Prediction performance of TEM-AID in the test set D.

	AUC (95%CI)	SEN (95%CI)	SPE (95%CI)	F1_score (95%CI)	Precision (95%CI)
DN	0.964(0.94,0.988)	0.81(0.733,0.887)	0.992(0.986,0.998)	0.862(0.804,0.908)	0.92(0.843,0.967)
IGA	0.986(0.978,0.993)	0.942(0.915,0.97)	0.937(0.919,0.955)	0.898(0.871,0.922)	0.859(0.814,0.896)
LN	0.986(0.972,1)	0.843(0.765,0.922)	0.995(0.991,1)	0.892(0.832,0.936)	0.946(0.867,0.985)
MCD	0.991(0.982,1)	0.924(0.878,0.969)	0.988(0.98,0.995)	0.924(0.885,0.953)	0.924(0.864,0.963)
MN	0.99(0.984,0.996)	0.977(0.96,0.994)	0.974(0.962,0.986)	0.961(0.943,0.975)	0.946(0.915,0.968)
MsPGN	0.947(0.911,0.983)	0.471(0.334,0.608)	0.981(0.972,0.99)	0.522(0.415,0.627)	0.585(0.421,0.737)
TBMN	0.964(0.938,0.99)	0.182(-0.046,0.41)	0.997(0.993,1)	0.25(0.073,0.524)	0.4(0.053,0.853)

DN = Diabetic Nephropathy. IgA = IgA Nephropathy. MCD = Minimal Change Disease. MN = Membranous Nephrosis. MsPGN = Mesangial Proliferative Glomerulonephritis. LN = Lupus Nephritis. TBMN= Thin Basement Membrane Nephropathy. TEM-AID = a novel TEM images based artificial intelligent diagnostic and analysis system.

© 2025 Ma P et al. *JAMA Network Open.*

2.8 eTable 8. Prediction performance of TEM-AID in the test set E.

	AUC (95%CI)	SEN (95%CI)	SPE (95%CI)	F1_score (95%CI)	Precision (95%CI)
DN	0.984(0.97,0.997)	0.793(0.711,0.876)	0.992(0.987,0.998)	0.849(0.786,0.899)	0.912(0.828,0.964)
IGA	0.991(0.986,0.997)	0.965(0.945,0.985)	0.949(0.933,0.965)	0.928(0.906,0.947)	0.894(0.856,0.925)
LN	0.983(0.966,1)	0.784(0.69,0.878)	0.997(0.993,1)	0.859(0.789,0.913)	0.951(0.863,0.99)
MCD	0.996(0.993,0.999)	0.955(0.922,0.987)	0.99(0.983,0.996)	0.948(0.918,0.97)	0.942(0.893,0.973)
MN	0.994(0.989,0.998)	0.978(0.962,0.994)	0.977(0.966,0.988)	0.964(0.947,0.977)	0.951(0.922,0.972)
MsPGN	0.973(0.96,0.987)	0.635(0.504,0.765)	0.985(0.977,0.992)	0.66(0.558,0.752)	0.688(0.537,0.813)
TBMN	0.984(0.965,1)	0.438(0.194,0.681)	0.998(0.995,1)	0.56(0.349,0.756)	0.778(0.4,0.972)

DN = Diabetic Nephropathy. IgA = IgA Nephropathy. MCD = Minimal Change Disease. MN = Membranous Nephrosis. MsPGN = Mesangial Proliferative Glomerulonephritis. LN = Lupus Nephritis. TBMN= Thin Basement Membrane Nephropathy. TEM-AID = a novel TEM images based artificial intelligent diagnostic and analysis system.

eTable 9. Performance of glomerular basement membrane segmentation

subtype	mIoU	Dice	Accuracy
DN	0.806	0.9	0.92
IGA	0.812	0.912	0.92
LN	0.78	0.853	0.89
MCD	0.899	0.886	0.96
MN	0.801	0.838	0.91
MsPGN	0.871	0.874	0.93
TBMN	0.842	0.892	0.83
**Total**	**0.835**	**0.874**	**0.91**

© 2025 Ma P et al. *JAMA Network Open.*

2.10 eTable 10. Professional training and working experiences of pathology experts

	Pathologists 1	Pathologists 2	Pathologists 3	Pathologists 4
Degree	Bachelor	Bachelor	Bachelor	Bachelor
Professional training in histopathological diagnosis, year	1	1	1	2
Professional training in renal pathology, year	3	3	3	3
Working experience in renal pathology, year	5	6	6	9
Working experience in electron microscopy diagnosis, year	5	6	6	9

© 2025 Ma P et al. *JAMA Network Open.*

2.11 eTable 11. Human-AI test results

Diseases	Pathologist 1	Pathologist 2	Pathologist 3	Pathologist 4	TEM-AID
Diabetic Nephropathy	12(52.1%)	13(56.5%)	15(65.2%)	14(60.8%)	16(69.5%)
IgA Nephropath	110(70.9%)	96(61.9%)	132(85.1%)	130(83.8%)	142(91.6%)
Thin Glomerular Basement Membrane Disease	5(71.4%)	5(71.4%)	6(85.7%)	10(100%)	2(28.5%)
Systemic Lupus Erythematosus LN	15(51.7%)	15(51.7%)	20(68.9%)	6(85.7%)	22(75.8%)
Minimal Change Disease	64(73.5%)	58(66.6%)	68(78.1%)	72(82.7%)	84(96.5%)
Mesangial proliferative-like glomerular lesions	14(58.3%)	13(54.1%)	19(79.1%)	19(79.1%)	18(75.0%)
Membranous Nephroitis	86(66.6%)	90(69.7%)	96(74.4%)	101(78.2%)	118(91.4%)
Total	306(67.4%)	290(63.8%)	356(78.4%)	363(79.9%)	402(88.5%)

TEM-AID = a novel TEM images based artificial intelligent diagnostic and analysis system.

3. Supplementary Figures

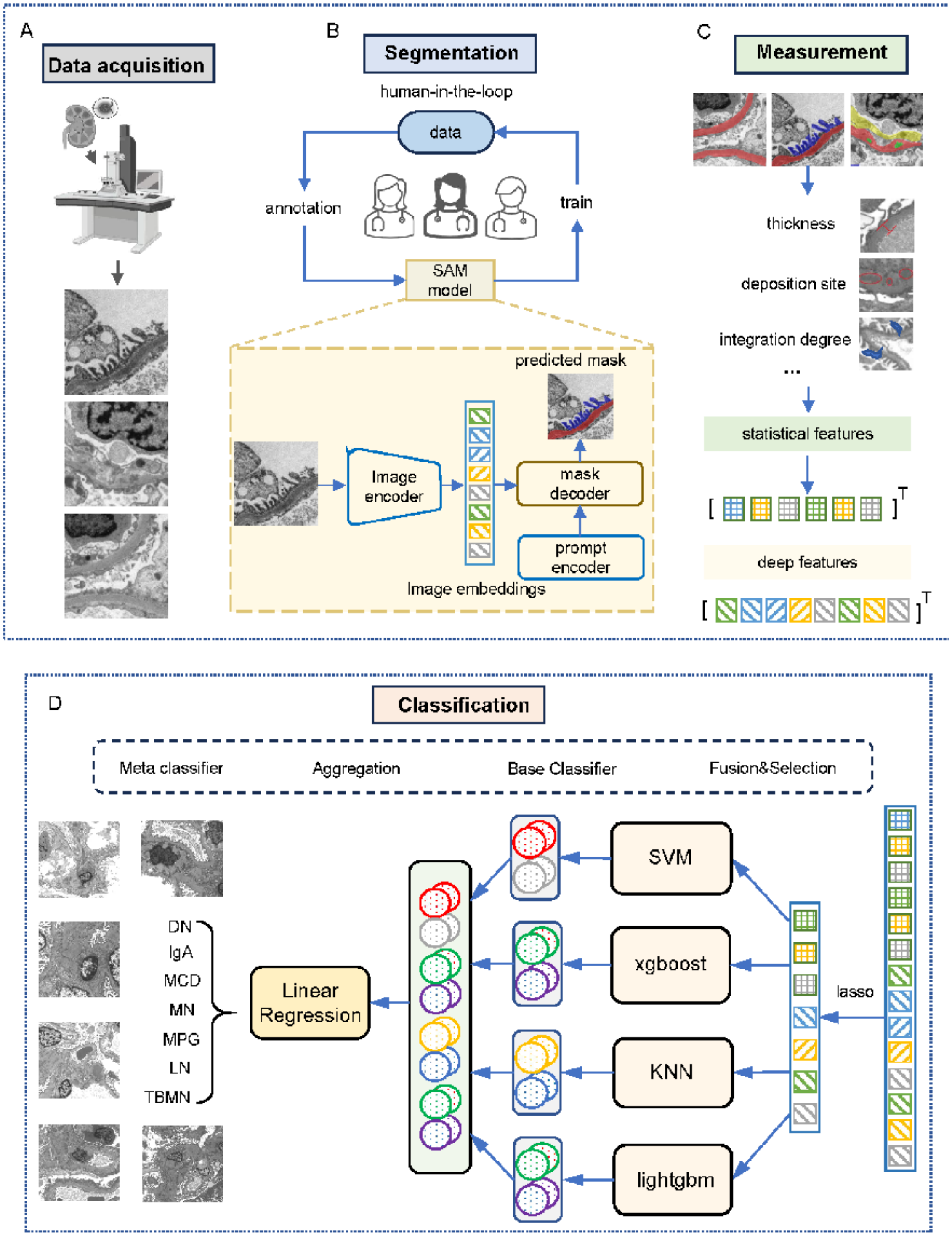

eFigure 1. Workflow of the proposed TEM-AID and study design. TEM-AID system consisting of Data acquisition, segmentation, measurement and classification modules. The

© 2025 Ma P et al. *JAMA Network Open.*

Segmentation module, which leverages an advanced framework termed TemNet. TemNet innovatively combines the YOLO-v8 detection model with the SAM segmentation model, further enhanced by integrating a human-in-the-loop mechanism. The measurement modules could be further divided into measuring the thickness of the basement membrane, assessing the fusion degree of podocytes, and determining the location of electron-dense deposits. The classification modules was divided into two stages: feature fusion and selection stage. In the feature fusion and selection stage, we refined the output of the TemNet model from the segmentation task to obtain DL features. In the classification stage, we proposed a stacking classifier consisting of a meta-classifier and four base classifiers.

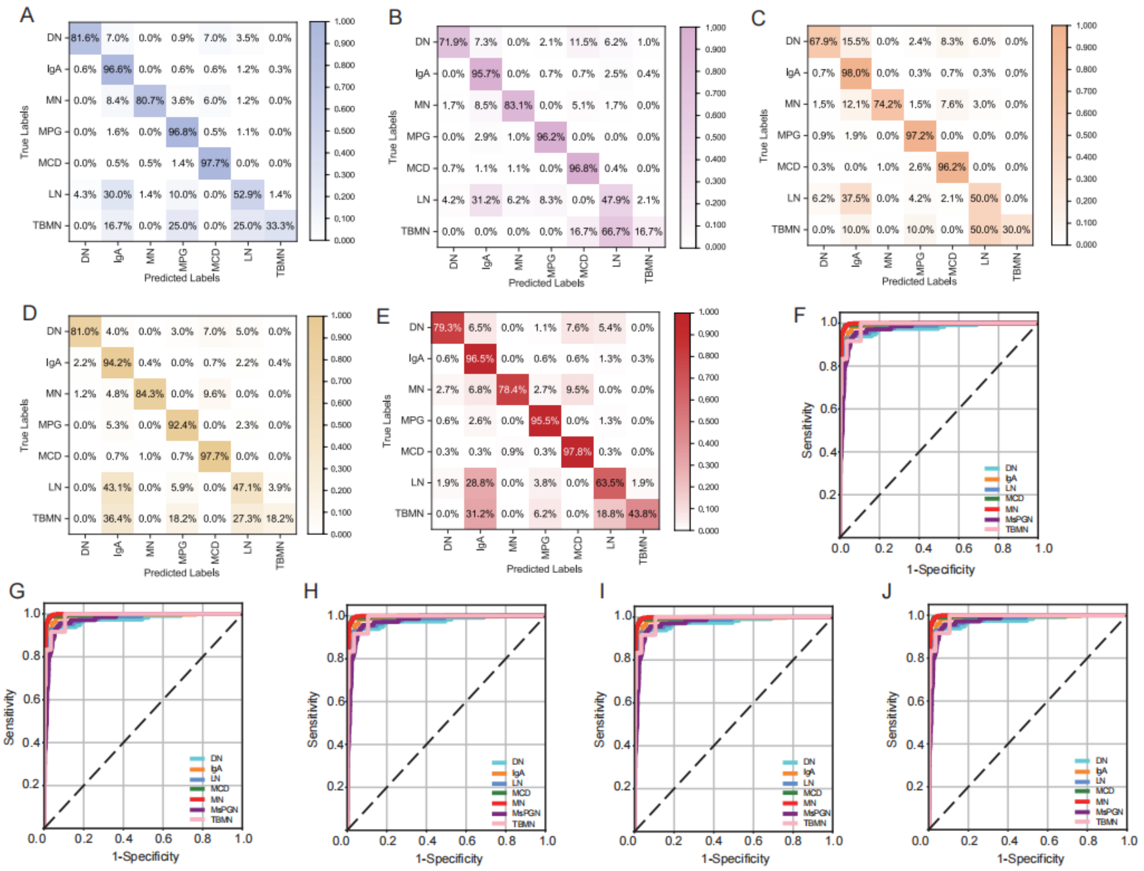

eFigure 2. A-E, Confusion Matrix of five external test datasets. The ROC analysis of TED-AID system in the (F) Test set A:Fujian, (G)Test set B:Gaozhou, (H) Test set C:Handan, (I) Test set D: Dongguan, (J) Test set E:Zhongshan (five centers). DN, Diabetic Nephropathy. IgA, IgA Nephropathy. MCD, Minimal Change Disease. MN, Membranous Nephrosis. MsPGN, Mesangial Proliferative Glomerulonephritis. LN,

© 2025 Ma P et al. *JAMA Network Open.*

Lupus Nephritis. TBMN, Thin Basement Membrane Nephropathy.

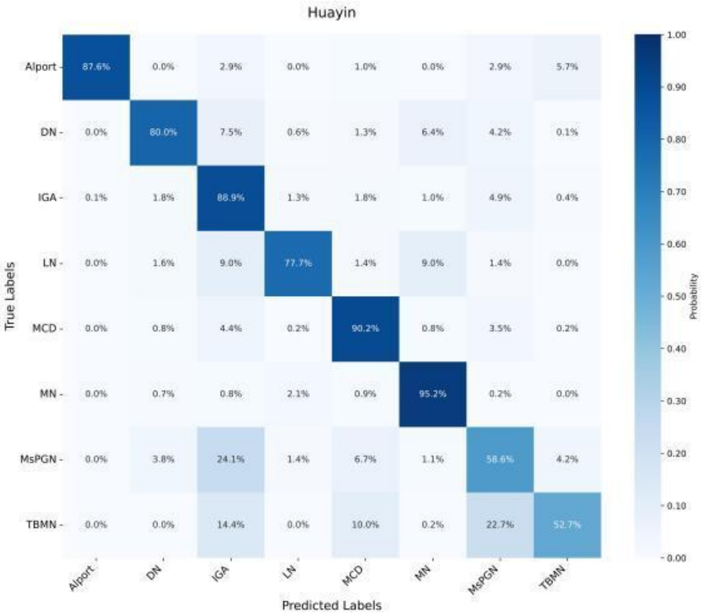

eFigure 3. Eight-class confusion matrices for TEM-AID performance on Huayin datasets. DN = Diabetic Nephropathy. IgA = IgA Nephropathy. MCD = Minimal Change Disease. MN = Membranous Nephrosis. MsPGN = Mesangial Proliferative Glomerulonephritis. LN = Lupus Nephritis. TBMN= Thin Basement Membrane Nephropathy. Alport = Alport syndrome.

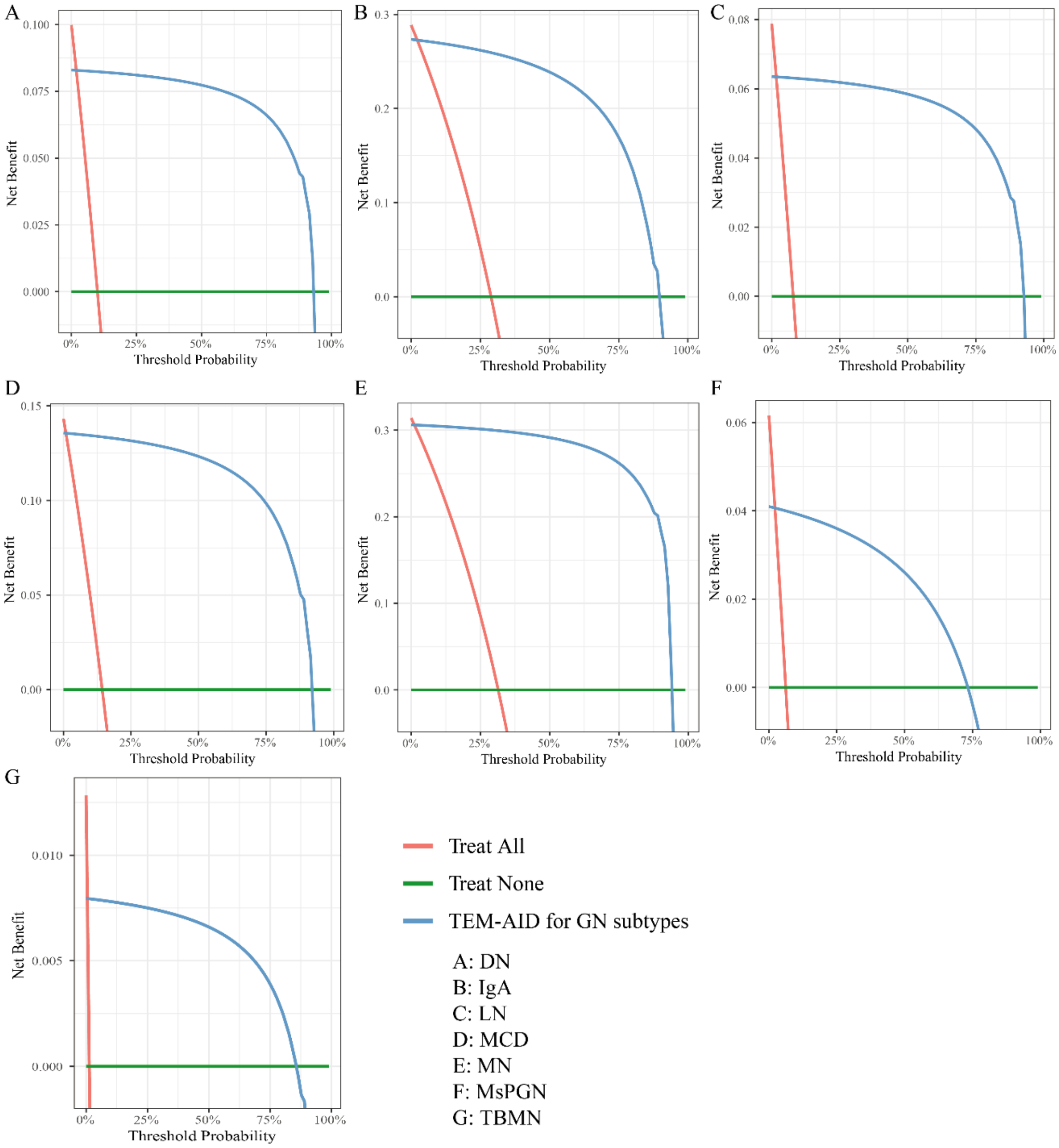

eFigure 4. DCA Curve of TEM-AID over seven subtypes of glomerulonephritis in the validation set. DN = Diabetic Nephropathy. IgA = IgA Nephropathy. MCD = Minimal Change Disease. MN = Membranous Nephrosis. MsPGN = Mesangial Proliferative Glomerulonephritis. LN = Lupus Nephritis. TBMN= Thin Basement Membrane Nephropathy. TEM-AID = a novel TEM images based artificial intelligent diagnostic and analysis system.

© 2025 Ma P et al. *JAMA Network Open.*

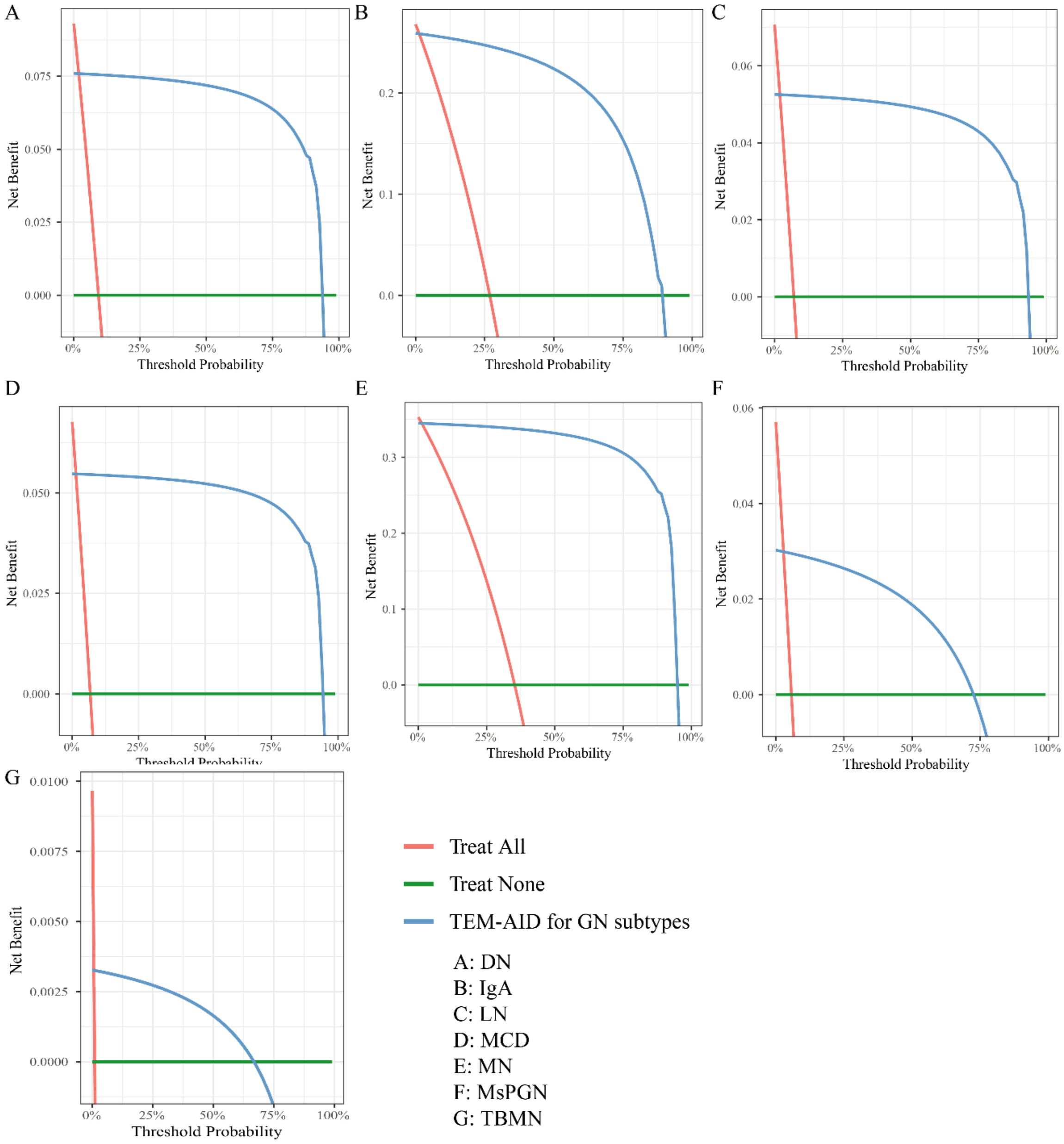

eFigure 5. DCA Curve of TEM-AID over seven subtypes of glomerulonephritis in the test set A.

DN = Diabetic Nephropathy. IgA = IgA Nephropathy. MCD = Minimal Change Disease. MN = Membranous Nephrosis. MsPGN = Mesangial Proliferative Glomerulonephritis. LN = Lupus Nephritis. TBMN= Thin Basement Membrane Nephropathy. TEM-AID = a novel TEM images based artificial intelligent diagnostic and analysis system.

© 2025 Ma P et al. *JAMA Network Open.*

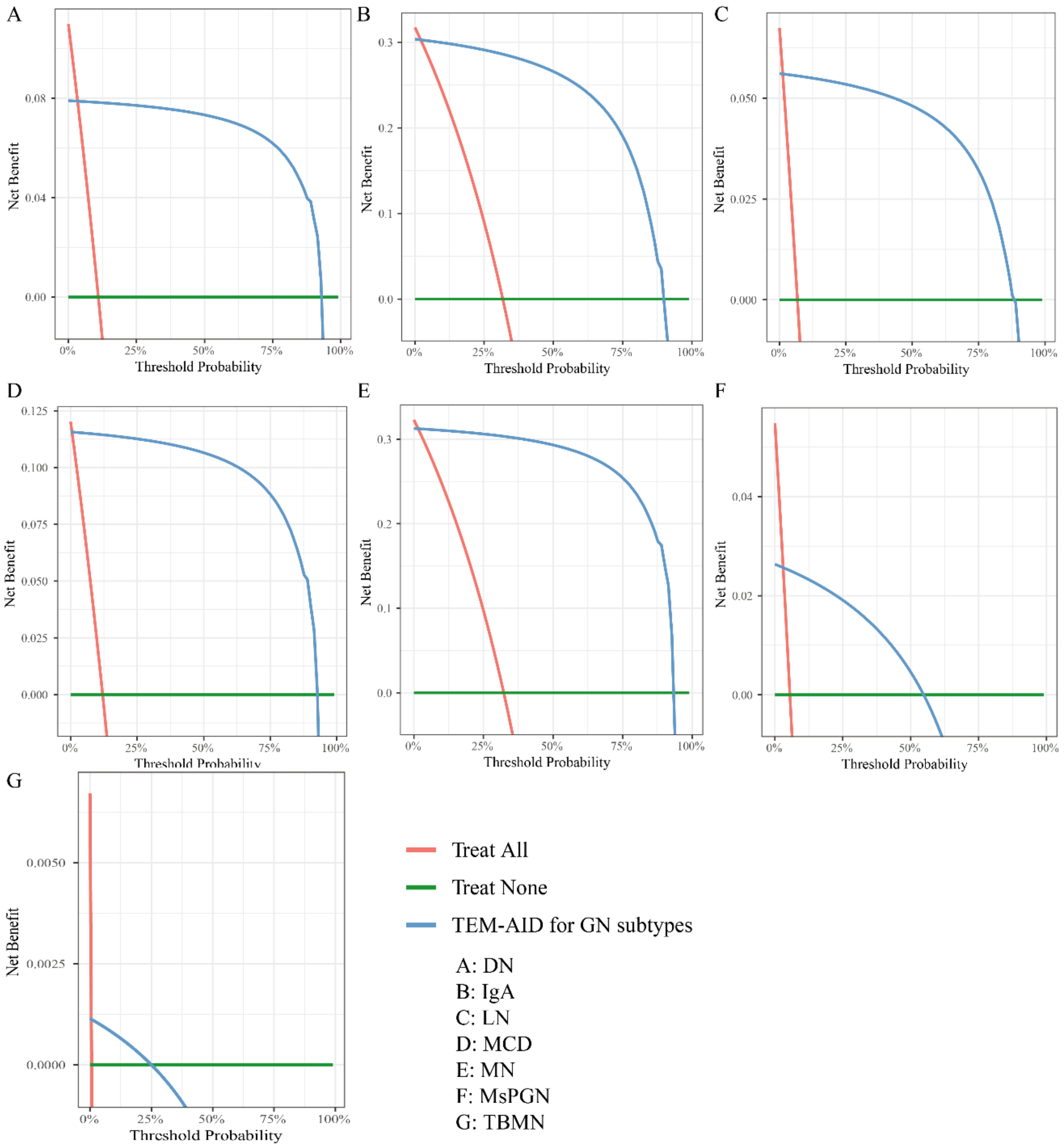

eFigure 6. DCA Curve of TEM-AID over seven subtypes of glomerulonephritis in the test set B.

DN = Diabetic Nephropathy. IgA = IgA Nephropathy. MCD = Minimal Change Disease. MN = Membranous Nephrosis. MsPGN = Mesangial Proliferative Glomerulonephritis. LN = Lupus Nephritis. TBMN= Thin Basement Membrane Nephropathy. TEM-AID = a novel TEM images based artificial intelligent diagnostic and analysis system.

© 2025 Ma P et al. *JAMA Network Open.*

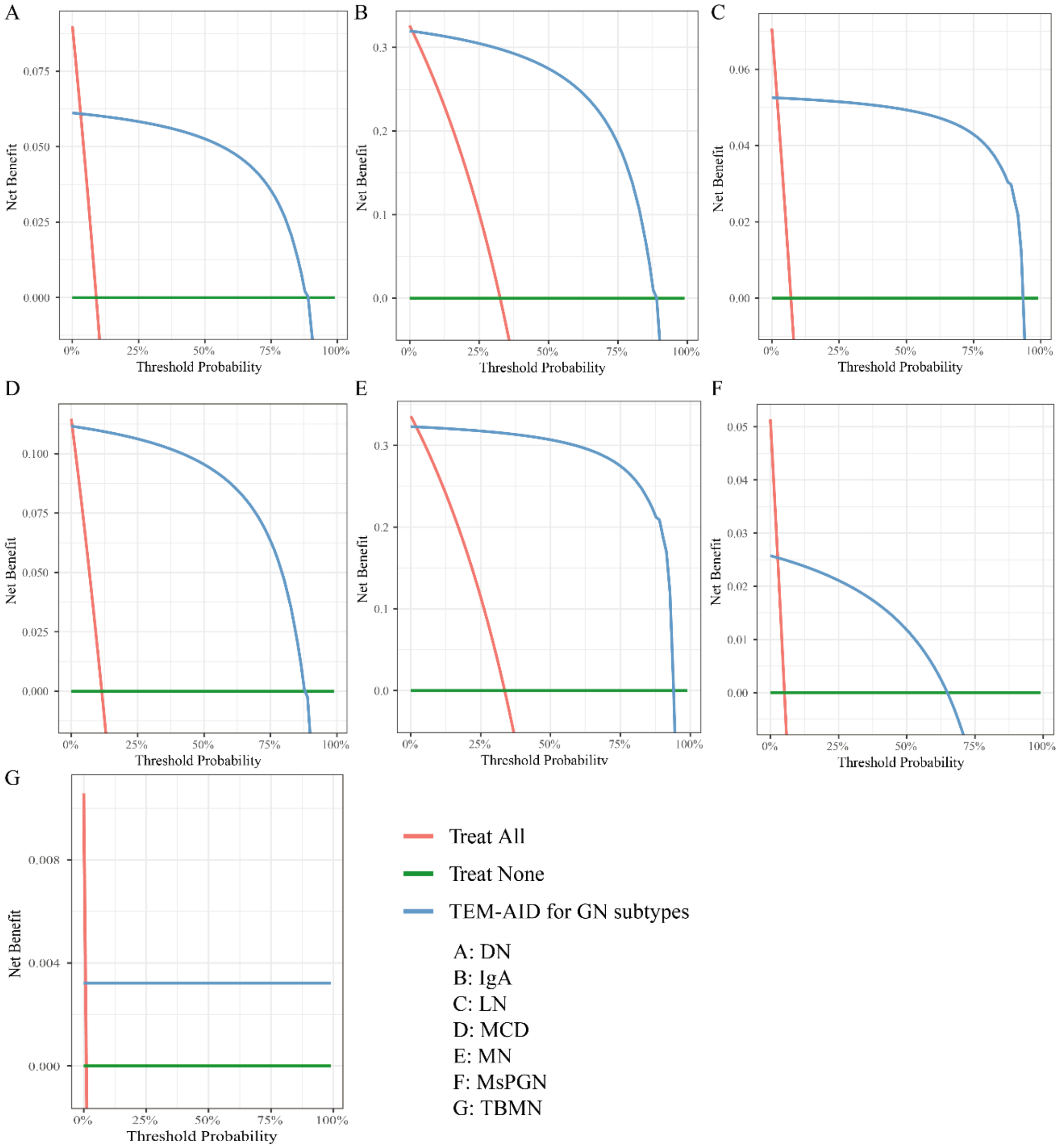

eFigure 7. DCA Curve of TEM-AID over seven subtypes of glomerulonephritis in the test set C.

DN = Diabetic Nephropathy. IgA = IgA Nephropathy. MCD = Minimal Change Disease. MN = Membranous Nephrosis. MsPGN = Mesangial Proliferative Glomerulonephritis. LN = Lupus Nephritis. TBMN= Thin Basement Membrane Nephropathy. TEM-AID = a novel TEM images based artificial intelligent diagnostic and analysis system.

© 2025 Ma P et al. *JAMA Network Open.*

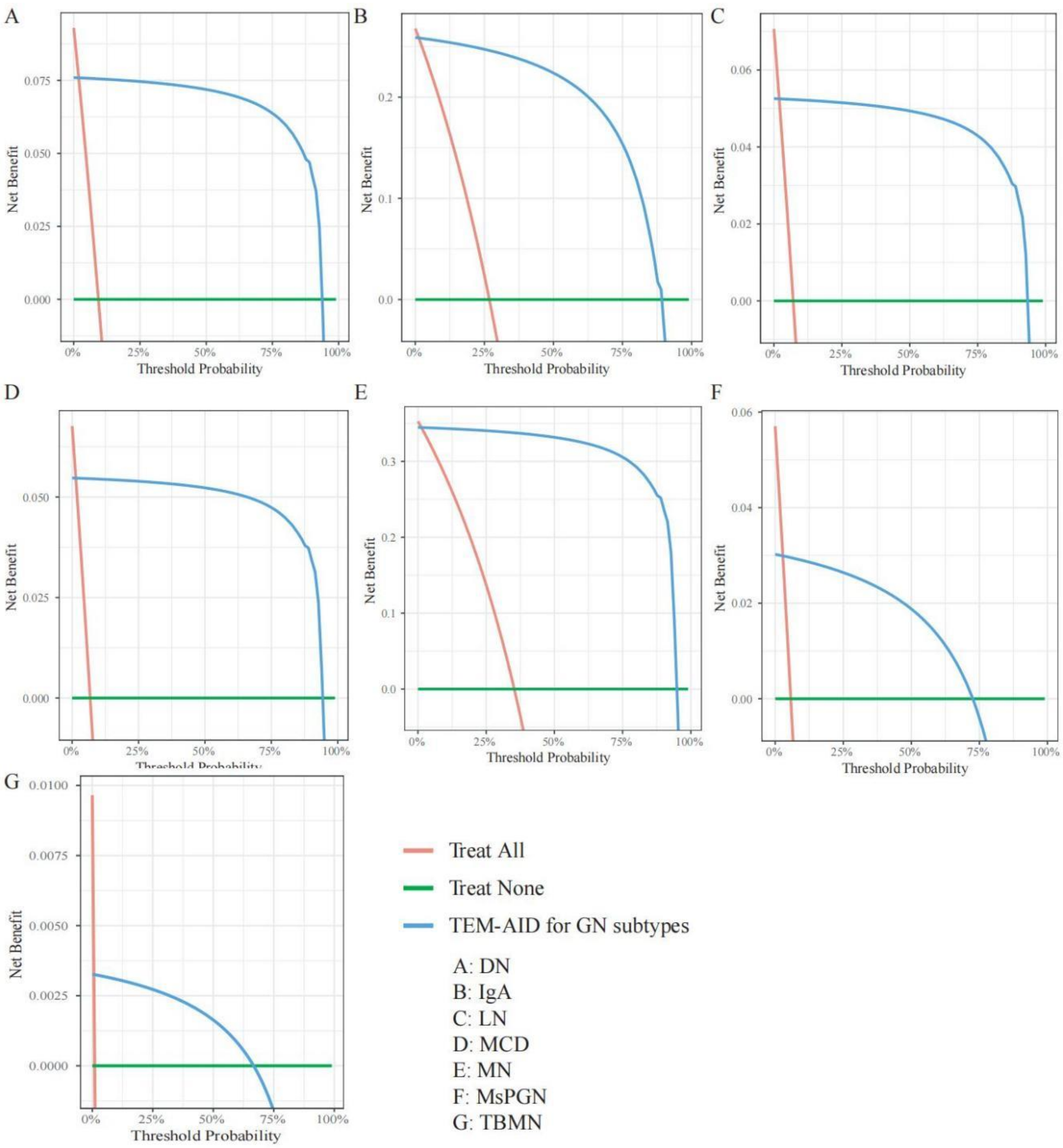

eFigure 8. DCA Curve of TEM-AID over seven subtypes of glomerulonephritis in the test set D.

DN = Diabetic Nephropathy. IgA = IgA Nephropathy. MCD = Minimal Change Disease. MN = Membranous Nephrosis. MsPGN = Mesangial Proliferative Glomerulonephritis. LN = Lupus Nephritis. TBMN= Thin Basement Membrane Nephropathy. TEM-AID = a novel TEM images based artificial intelligent diagnostic and analysis system.

© 2025 Ma P et al. *JAMA Network Open*.

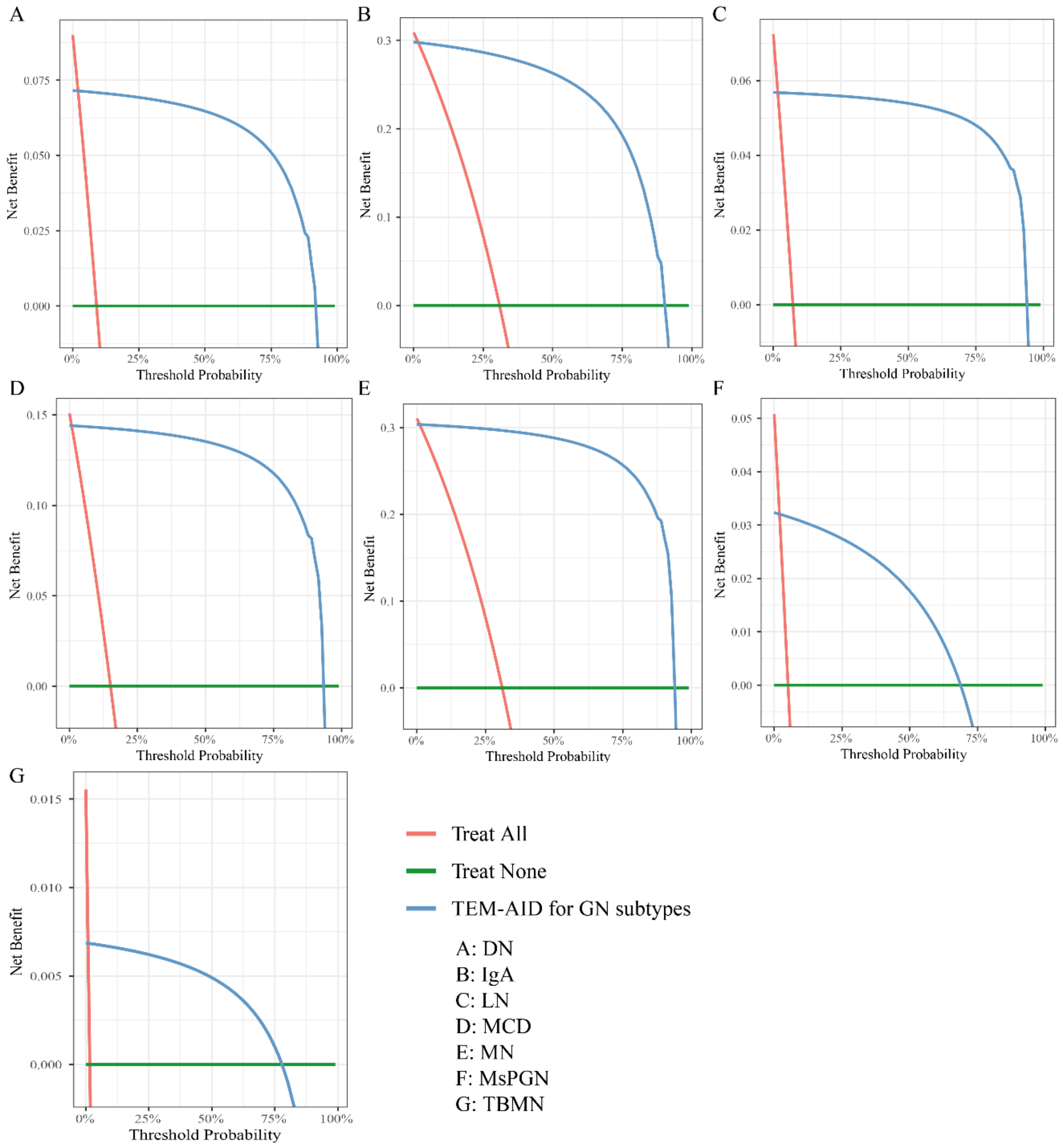

eFigure 8. DCA Curve of TEM-AID over seven subtypes of glomerulonephritis in the test set E.

DN = Diabetic Nephropathy. IgA = IgA Nephropathy. MCD = Minimal Change Disease. MN = Membranous Nephrosis. MsPGN = Mesangial Proliferative Glomerulonephritis. LN = Lupus Nephritis. TBMN= Thin Basement Membrane Nephropathy. TEM-AID = a novel TEM images based artificial intelligent diagnostic and analysis system.

© 2025 Ma P et al. *JAMA Network Open.*

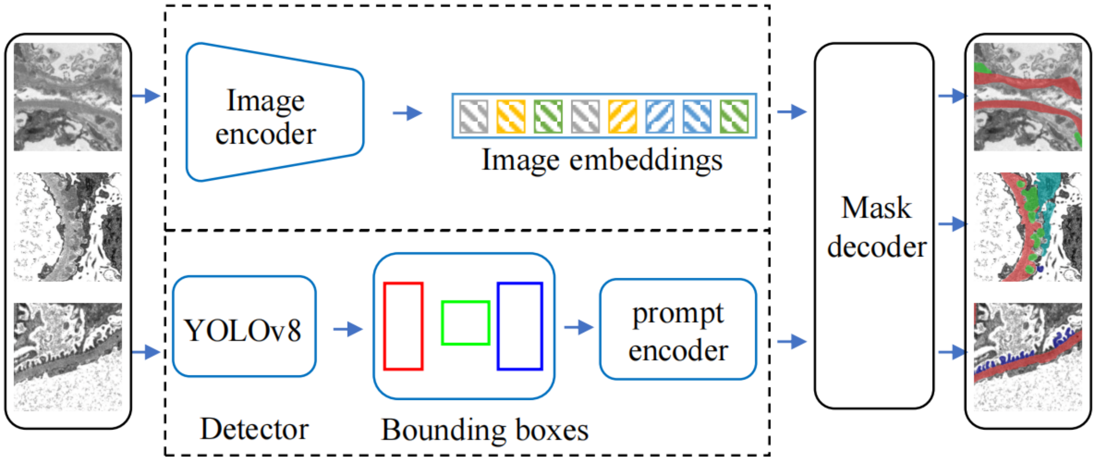

eFigure 9. Architecture of YOLOv8-SAM

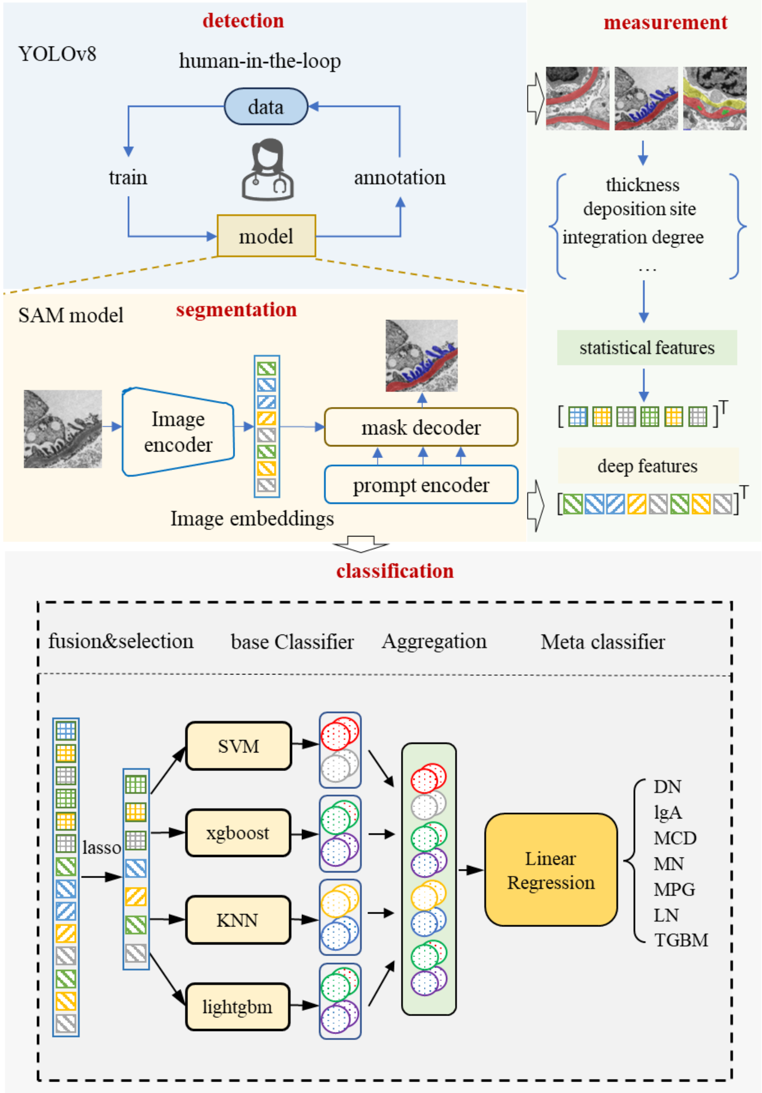

eFigure 10. Architecture of TEM-AID

© 2025 Ma P et al. *JAMA Network Open.*

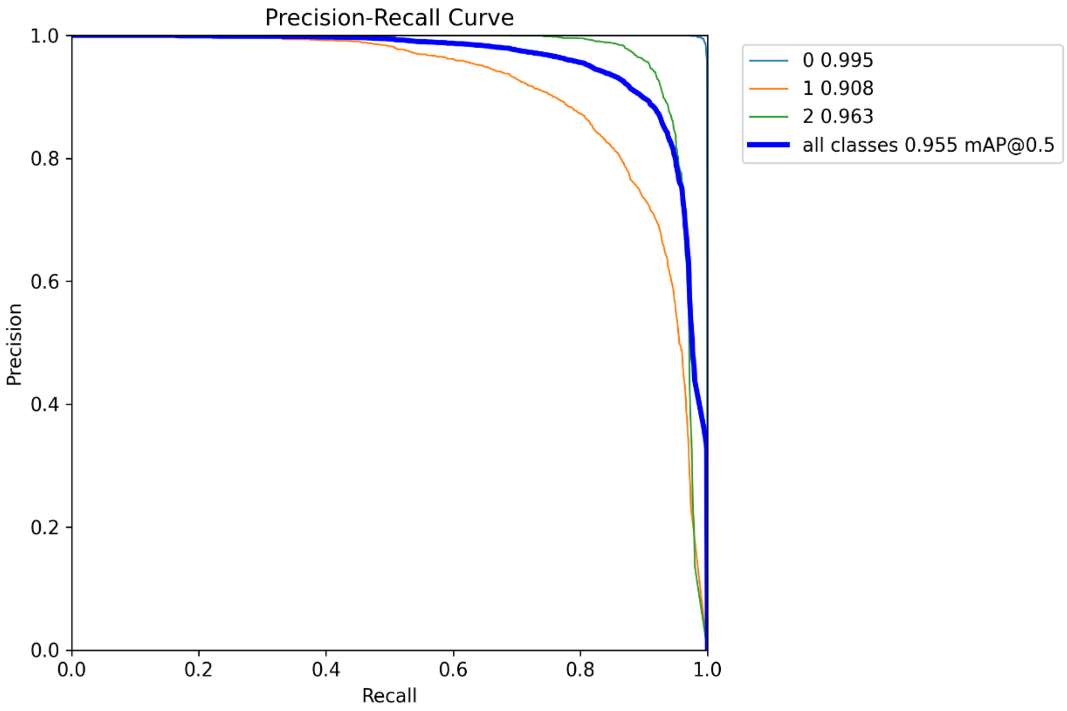

eFigure 11. Box Precision-Recall Curve

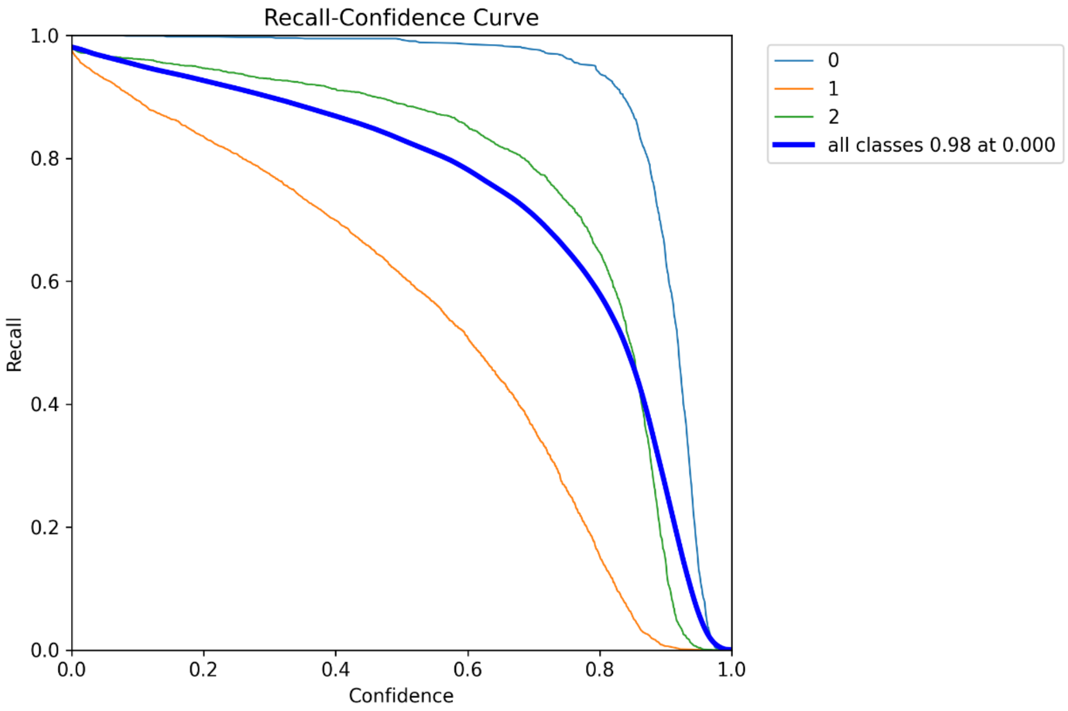

eFigure 12. Box Recall-Confidence Curve

© 2025 Ma P et al. *JAMA Network Open.*

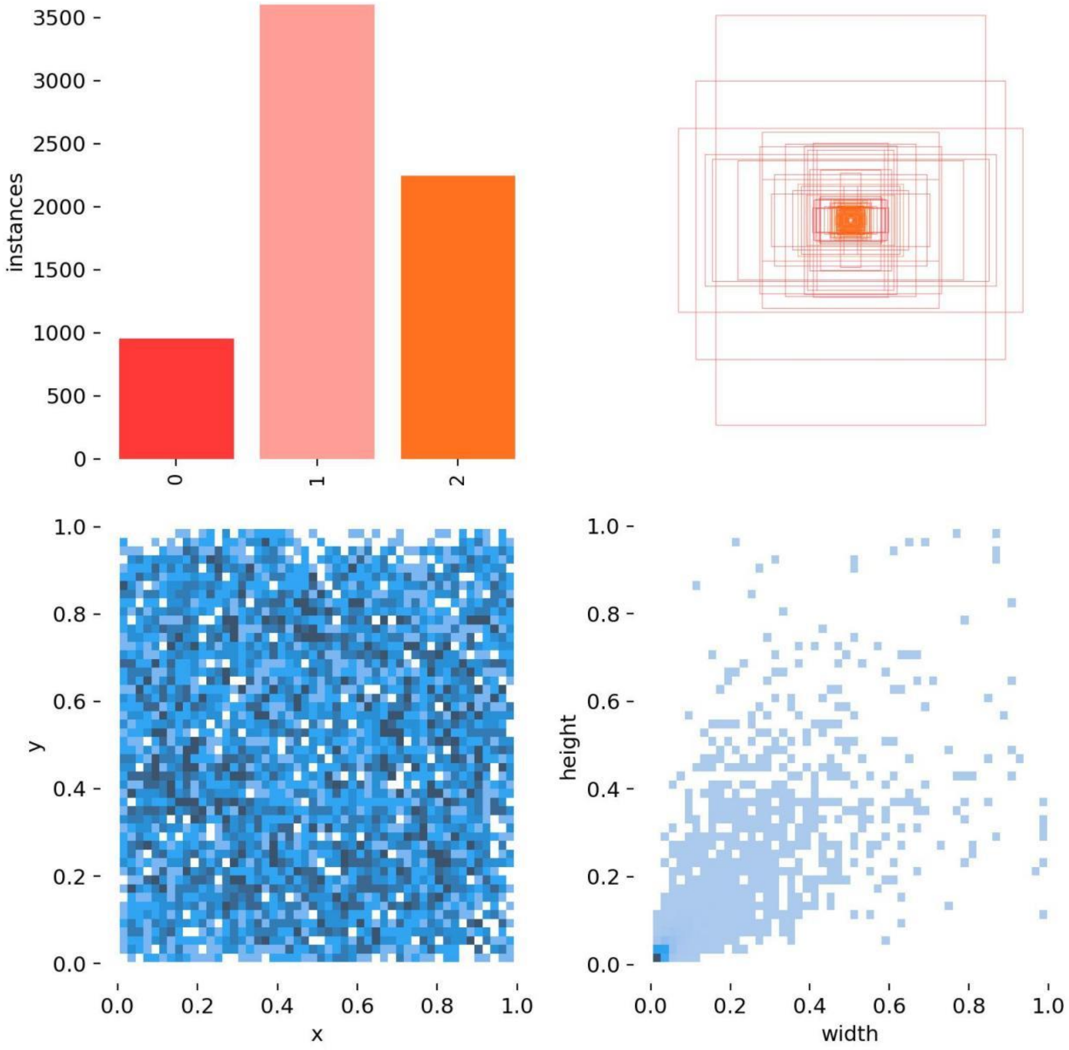

eFigure 13. Labels Distribution

© 2025 Ma P et al. *JAMA Network Open.*

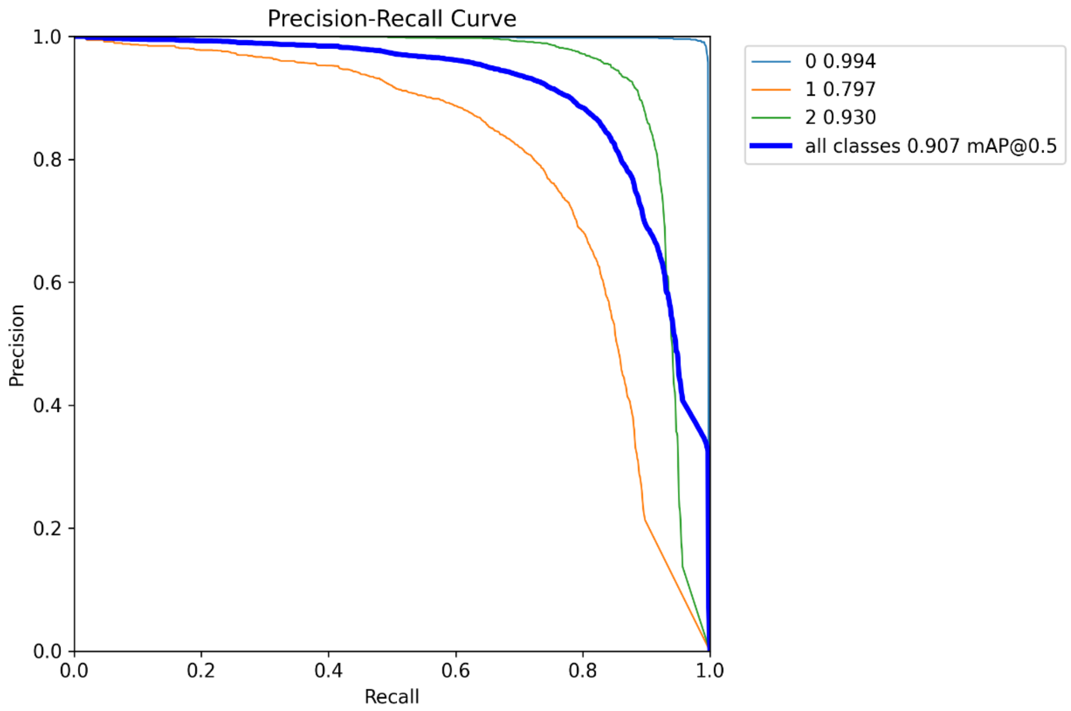

eFigure 14. Mask Precison-Recall Curve

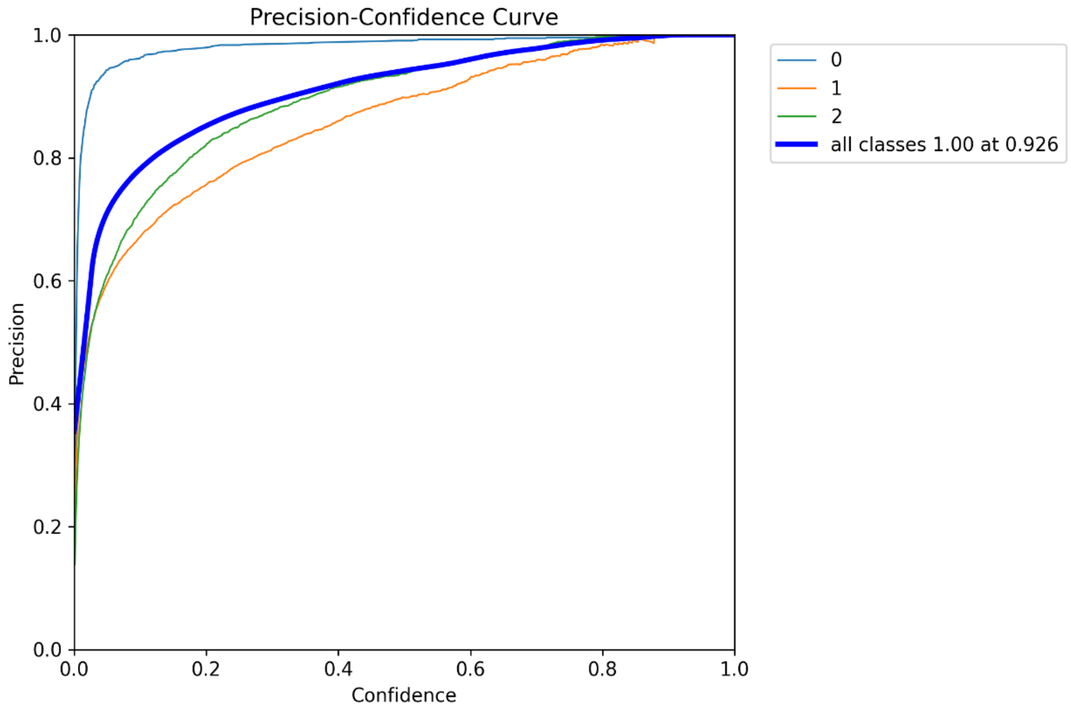

eFigure 15. Mask Precision-Confidence Curve

© 2025 Ma P et al. *JAMA Network Open*.

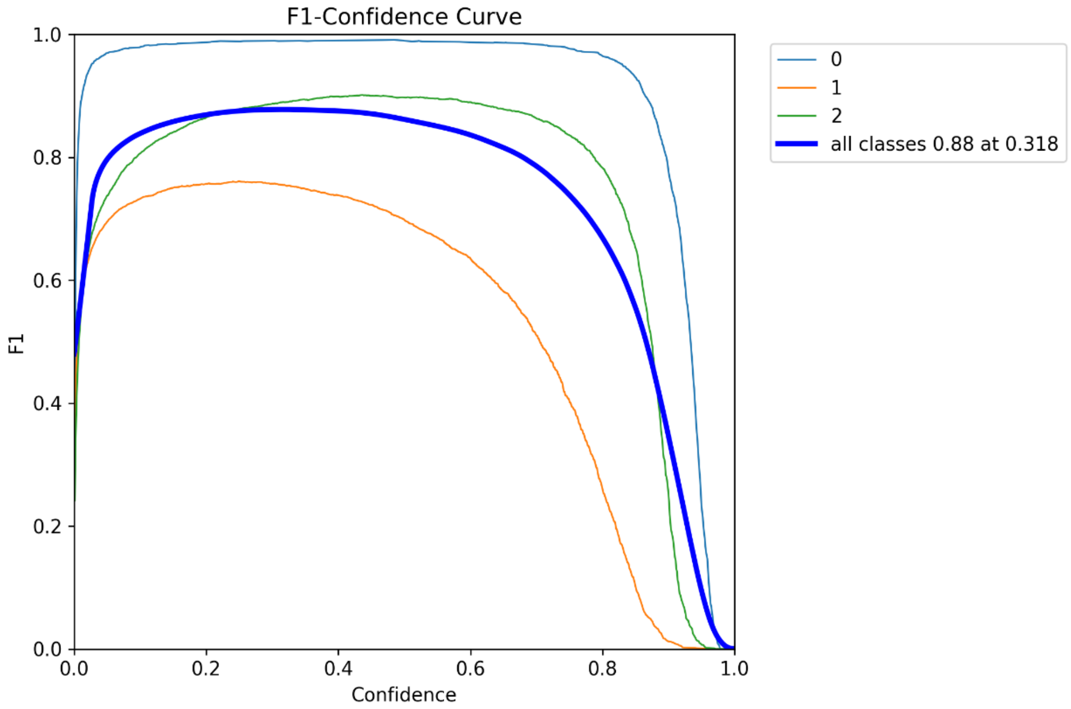

eFigure 16. Mask F1-Confidence Curve

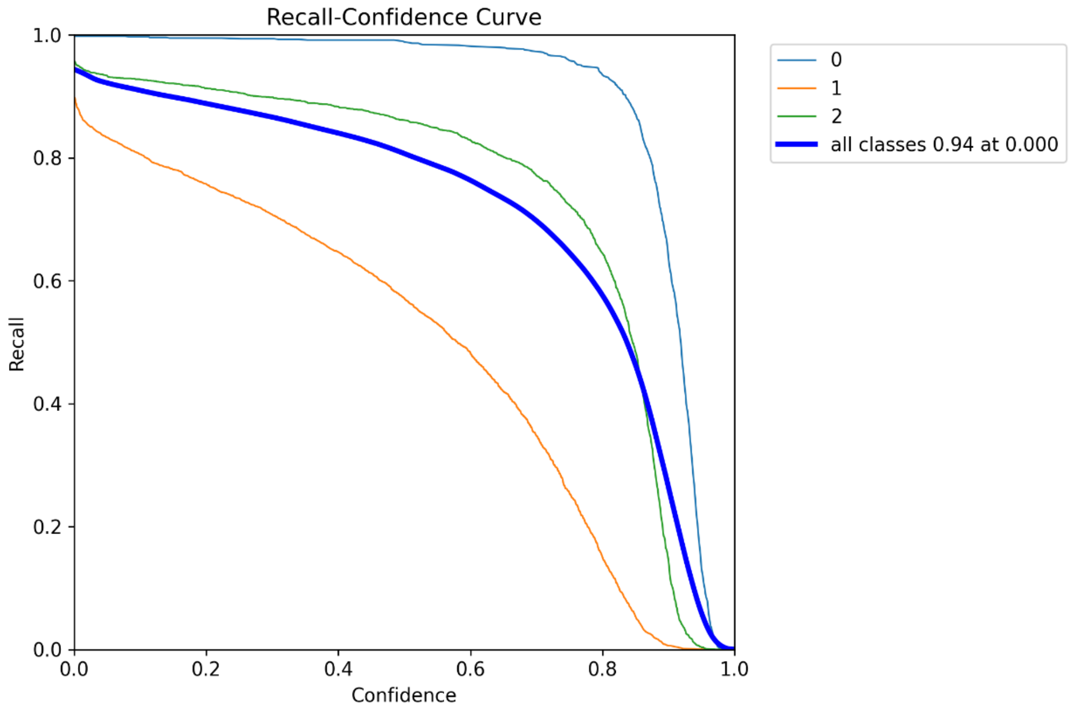

eFigure 17. Mask Recall-Confidence Curve

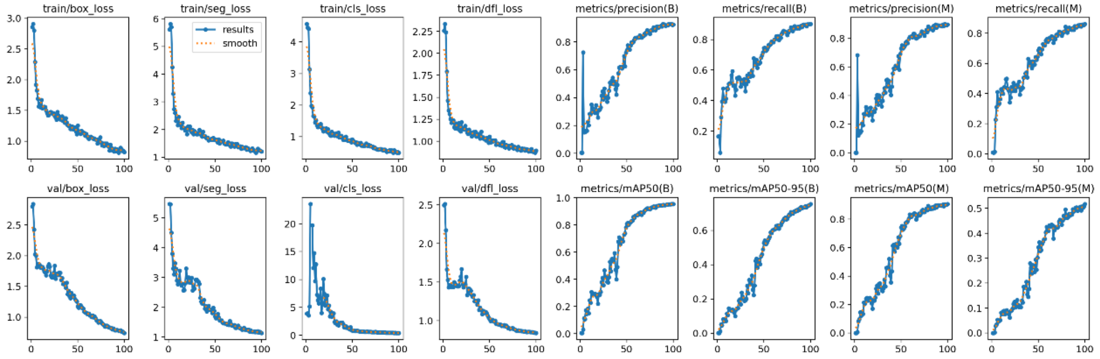

eFigure 18. Training results

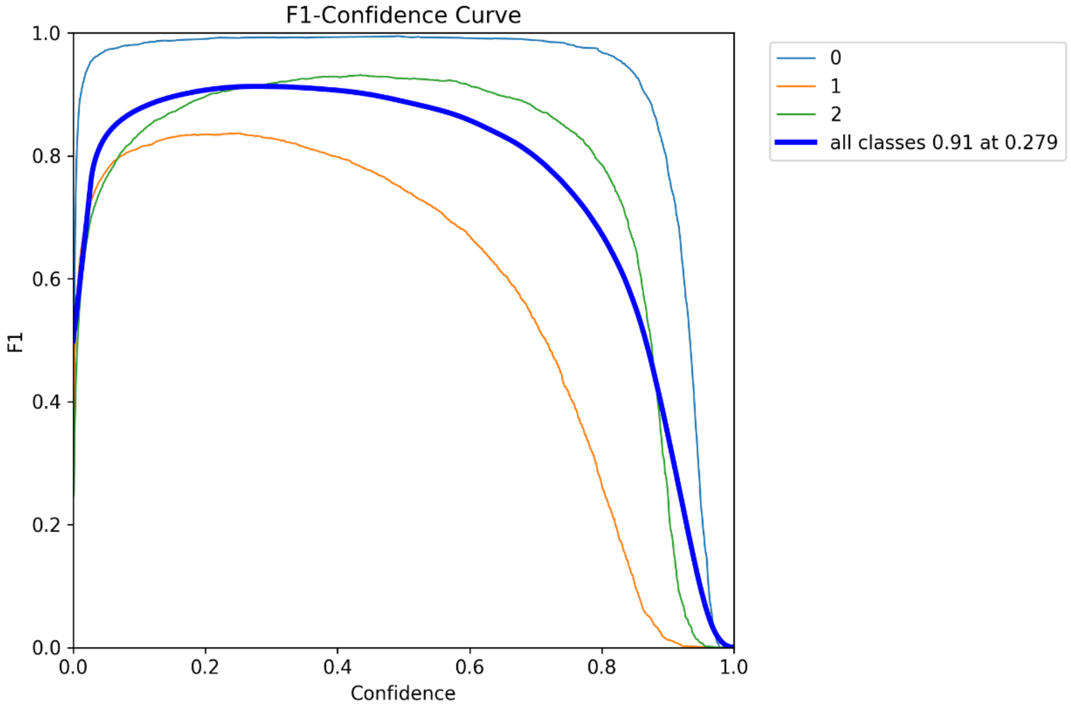

eFigure 19. Box F1-Confidence Curve

© 2025 Ma P et al. *JAMA Network Open*.

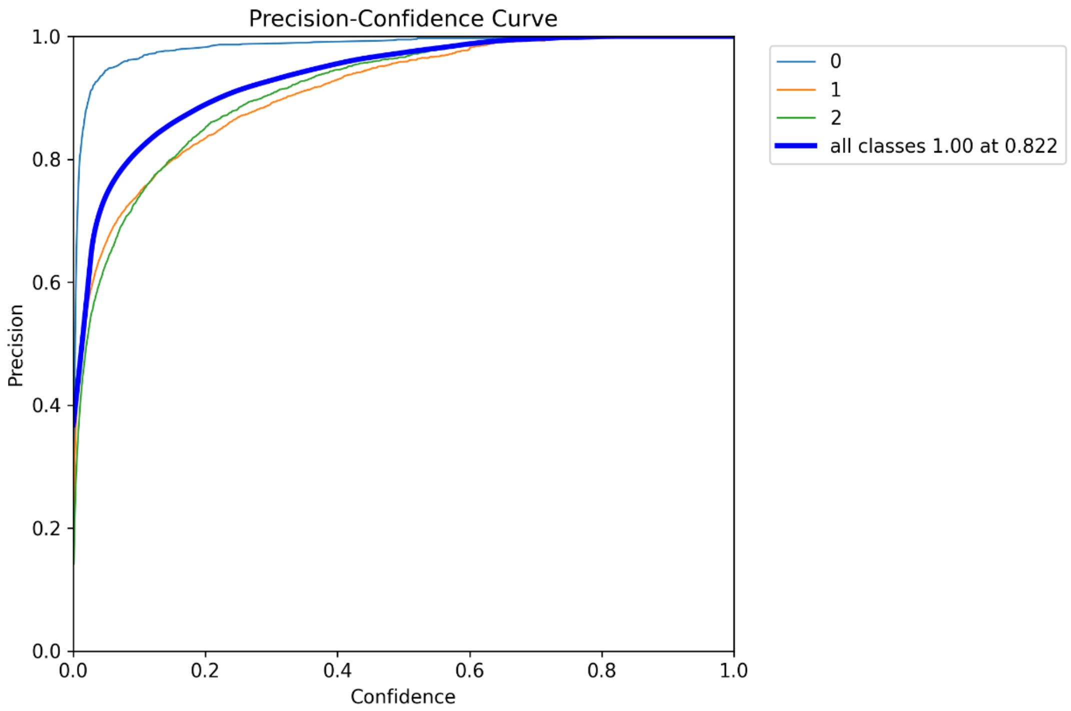

eFigure 20. Box Precision-Confidence Curve

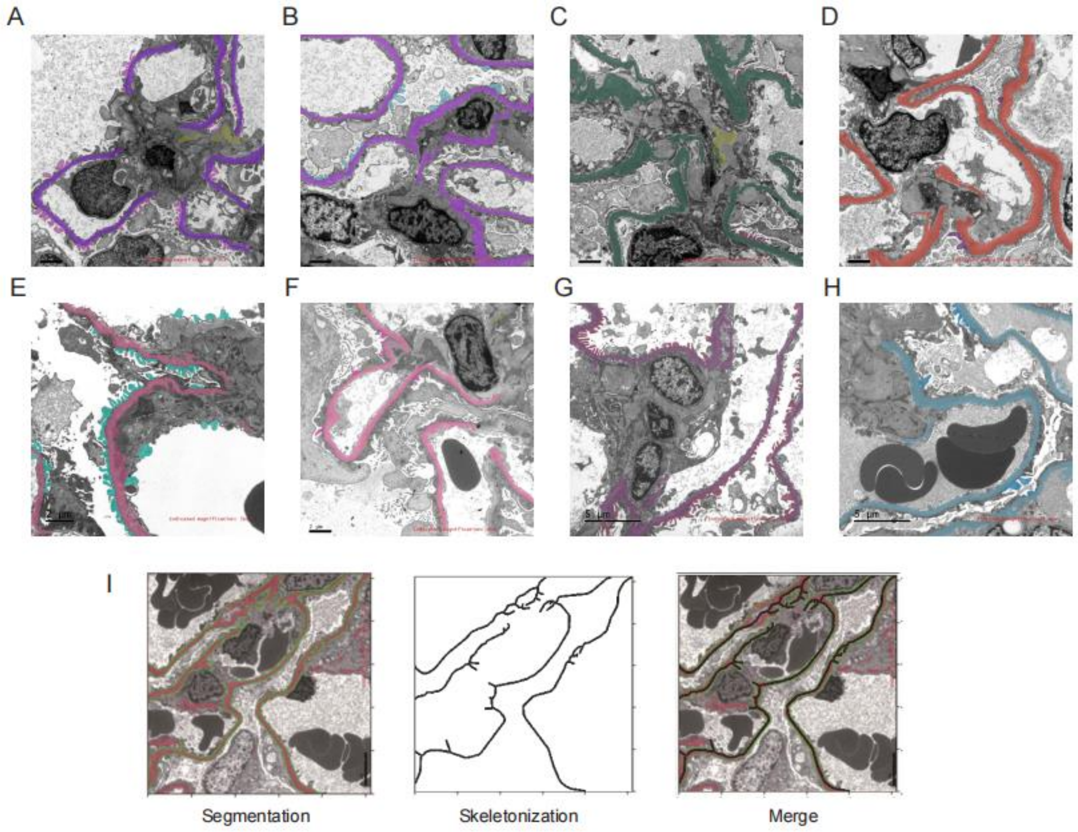

eFigure 21. A-H, TEM-AID segmentation of glomerular basement membranes, electron-dense materialand and foot processes. I, Precise segmentation, skeletonization, and thickness measurement of glomerular basement membrane.

© 2025 Ma P et al. *JAMA Network Open*.

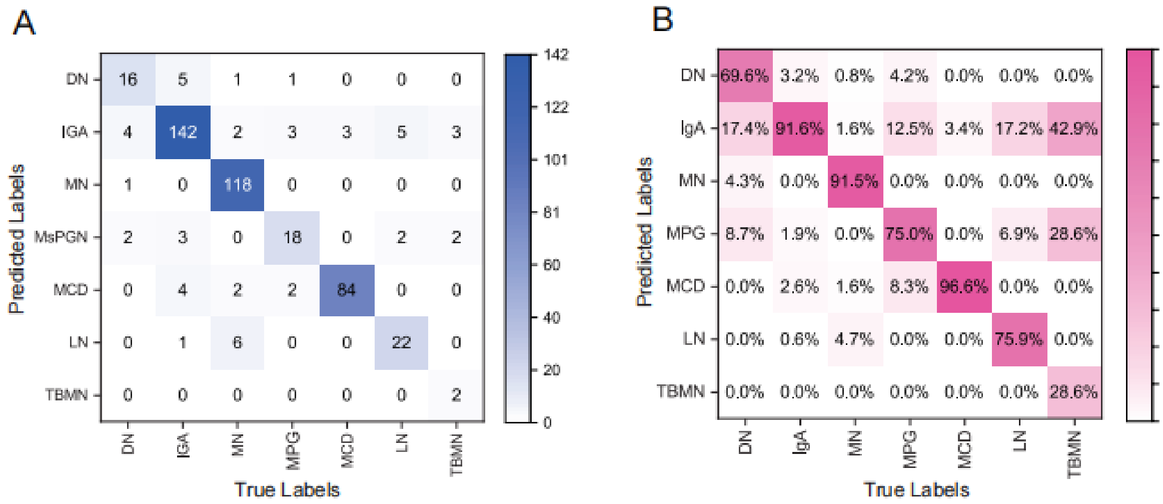

eFigure 22. The results of human-AI test. A-B, The absolute (left image) and relative (right image) confusion matrices of TED-AID in human-AI test. DN, Diabetic Nephropathy. IgA, IgA Nephropathy. MCD, Minimal Change Disease. MN, Membranous Nephrosis. MsPGN, Mesangial Proliferative Glomerulonephritis. LN, Lupus Nephritis. TBMN, Thin Basement Membrane Nephropathy. AUC, area under the curve.

eAppendix: Detailed Explanation of TEM-AID system

This document provides clear explanations and definitions for key terms used in the TEM-AID system to enhance accessibility and readability for readers with varying levels of technical expertise.

1. Simple Introduction to TEM-AID Workflow

Imagine TEM-AID as a smart assistant for doctors analyzing kidney tissue images:

Step 1: Finding the Important Bits (Segmentation)

The system looks at a black-and-white microscope image of kidney tissue (a TEM image).

It automatically identifies and outlines three key things:

The Basement Membrane: A thin wall-like structure.

Foot Processes: Tiny finger-like projections on certain kidney cells.

© 2025 Ma P et al. *JAMA Network Open.*

Electron-Dense Deposits: Clumps of material that shouldn't be there (often immune system debris).

Step 2: Measuring the Important Bits (Measurement)

Once it knows where these things are, TEM-AID starts measuring them:

Basement Membrane Thickness: It carefully measures how thick the wall-like membrane is at many points and calculates an average thickness.

Foot Process Fusion: It checks how many of those tiny finger-like projections are stuck together or "fused" and gives this a rating (like "a little fused" or "a lot fused").

Deposit Location: It figures out exactly where the unwanted clumps are located.

Step 3: Putting It All Together (Classification)

TEM-AID takes all the information it found (the outlines and measurements) and combines it.

It uses this combined information to figure out what type of kidney disease (glomerular disease) the patient likely has. It can distinguish between 7 common subtypes.

Essentially, it asks: "Based on how thick the wall is, how many fingers are fused, and where the clumps are, what disease pattern does this match?"

The Big Picture:

Speed: This whole process happens very quickly (around 1-3 seconds per image analysis).

Accuracy: The system was trained on a huge number of images and checked by doctors. Tests showed it can identify diseases very accurately, often matching or even exceeding the accuracy of human specialists.

Help for Doctors: TEM-AID acts like a powerful tool for pathologists (doctors who analyze tissue samples). It gives them precise measurements and a suggested diagnosis based on the image, helping them work faster and make more confident decisions.

In short: TEM-AID looks at microscope pictures of kidney tissue, automatically finds and measures key structures, and uses that information to help doctors figure out what kidney disease a patient has.

© 2025 Ma P et al. *JAMA Network Open.*

2. Glossary of Key Terms for TEM-AID System

Artificial Intelligence (AI)

Definition: AI refers to the simulation of human intelligence in machines that are programmed to think like humans and mimic their actions. In the context of TEM-AID, AI is used to analyze and interpret transmission electron microscopy (TEM) images for diagnosing glomerular diseases.

Example: The TEM-AID system uses AI to automatically identify and measure specific structures in TEM images, such as the glomerular basement membrane and foot processes.

Transmission Electron Microscopy (TEM)

Definition: TEM is a microscopy technique that uses a beam of electrons to create high-resolution images of thin samples. It is essential for visualizing ultrastructural details in biological tissues, including kidney biopsies.

Example: TEM images are used in the TEM-AID system to provide detailed views of the glomerular basement membrane, foot processes, and electron-dense deposits, which are critical for diagnosing glomerular diseases.

Glomerular Diseases

Definition: Glomerular diseases are conditions that affect the glomeruli, the tiny filtering units in the kidneys. These diseases can lead to chronic kidney disease (CKD) and include conditions such as IgA nephropathy, membranous nephropathy, and lupus nephritis.

Example: The TEM-AID system is designed to diagnose and classify seven common glomerular diseases, including diabetic nephropathy, IgA nephropathy, and minimal change disease.

Segmentation

Definition: In the context of image analysis, segmentation refers to the process of partitioning an image into multiple segments or regions to simplify and interpret the image more effectively.

Example: The TEM-AID system uses segmentation to precisely outline and measure the glomerular basement membrane, podocytes, and electron-dense deposits in TEM images.

Classification

Definition: Classification is the process of categorizing data into predefined groups or classes based on specific features or characteristics.

© 2025 Ma P et al. *JAMA Network Open.*

Example: The TEM-AID system classifies glomerular diseases into seven distinct subtypes based on the segmented and measured features from TEM images.

Human-in-the-Loop (HITL)

Definition: HITL is an approach in AI where human input is incorporated into the training process to improve the model's performance. It involves human experts reviewing and correcting the model's predictions, which are then used to further train the model.

Example: In the TEM-AID system, medical experts review and correct the initial segmentation results, providing valuable feedback that enhances the model's accuracy over time.

Ensemble Learning

Definition: Ensemble learning is a technique that combines multiple machine learning models to improve overall performance. It leverages the strengths of different algorithms to achieve better predictive accuracy.

Example: The TEM-AID system uses an ensemble learning approach, combining multiple classifiers (such as SVM, XGBoost, KNN, and LightGBM) to accurately classify glomerular diseases.

External Validation

Definition: External validation refers to the process of evaluating a model's performance on datasets that were not used during the training phase. This helps assess the model's generalizability and robustness.

Example: The TEM-AID system was validated on five independent external test cohorts to ensure its performance is consistent across different datasets and clinical settings.

Confusion Matrix

Definition: A confusion matrix is a table used to evaluate the performance of a classification model. It shows the number of true positives, false positives, true negatives, and false negatives.

Example: The TEM-AID system provides detailed confusion matrices for each external test cohort, demonstrating its high accuracy and robustness in classifying glomerular diseases.

ROC Curve and AUC

Definition: The Receiver Operating Characteristic (ROC) curve is a graphical

representation of a model's performance, plotting the true positive rate against the false positive rate. The Area Under the Curve (AUC) provides a single metric summarizing the model's overall performance.
Example: The TEM-AID system achieves high AUC values across all external test cohorts, indicating its excellent diagnostic accuracy and reliability.

© 2025 Ma P et al. *JAMA Network Open*.
