# Supplementary material for: AI-Based System for Analysis of Electron Microscope Images in Glomerular Disease
Source: JAMA Netw Open. 2025 Oct 7;8(10):e2534985. doi: 10.1001/jamanetworkopen.2025.34985 (PMC12505174; doi:10.1001/jamanetworkopen.2025.34985)
Supplement: Supplement 2. — Data Sharing Statement [file jamanetwopen-e2534985-s002.pdf]

Data Sharing Statement

Ma. AI-Based System for Analysis of Electron Microscope Images in Glomerular Disease. *JAMA Netw Open*. Published October 02, 2025. doi:10.1001/jamanetworkopen.2025.34985

Data
Data available: No

Additional Information
Explanation for why data not available: All articles in this manuscript are available from PubMed, Embase, CINAHL, and The Cochrane Library. The analysis codes are available from the corresponding author on reasonable request.
